# LTA1 and dmLT enterotoxin-based proteins activate antigen-presenting cells independent of PKA and despite distinct cell entry mechanisms

**DOI:** 10.1371/journal.pone.0227047

**Published:** 2020-01-13

**Authors:** Eduardo Valli, Robin L. Baudier, Amanda J. Harriett, Elizabeth B. Norton

**Affiliations:** Department of Microbiology & Immunology, Tulane University School of Medicine, New Orleans, LA, United States of America; Instituto Butantan, BRAZIL

## Abstract

Enterotoxin-based proteins are powerful manipulators of mucosal immunity. The A1 domain of heat-labile enterotoxin from *E*. *coli*, or LTA1, is a newer adjuvant from this family under investigation for intranasal vaccines. Although LTA1 has been examined in mouse vaccination studies, its ability to directly stimulate immune cells compared to related adjuvant proteins has not been well explored. Here, we perform the first rigorous examination of LTA1’s effect on antigen presenting cells (APC) using a human monocyte cell line THP-1. To better understand LTA1’s stimulatory effects, we compared it to dmLT, or LT-R192G/L211A, a related AB_5_ adjuvant in clinical trials for oral or parenteral vaccines. LTA1 and dmLT both activated APCs to upregulate MHC-II (HLA-DR), CD86, cytokine secretion (e.g., IL-1β) and inflammasome activation. The effect of LTA1 on surface marker changes (e.g., MHC-II) was highly dose-dependent whereas dmLT exhibited high MHC-II expression regardless of dose. In contrast, cytokine secretion profiles were similar and dose-dependent after both LTA1 and dmLT treatment. Cellular activation by LTA1 was independent of ganglioside binding, as pre-treatment with purified GM1 blocked the effect of dmLT but not LTA1. Unexpectedly, while activation of the inflammasome and cytokine secretion by LTA1 or dmLT was blocked by the protein kinase A inhibitor H89 (similar to previous reports), these responses were not inhibited by a more specific PKA peptide inhibitor or antagonist; thus Indicating that a novel and unknown mechanism is responsible for inflammasome activation and cytokine secretion by LT proteins. Lastly, LTA1 stimulated a similar cytokine profile in primary human monocytes as it did in THP1 cells, including IL-1β, IL-6, IL-8, MIP-1α, MIP-1β, and TNFα. Thus, we report that LTA1 protein programs a dendritic cell-like phenotype in APCs similar to dmLT in a mechanism that is independent of PKA activation and GM1 binding and entry.

## Introduction

Adjuvants are useful additives that promote vaccine induced immunity, however, only a few adjuvant compounds can be used at mucosal surfaces or to induce mucosal immunity. The best known mucosal adjuvants are enterotoxin proteins, including heat-labile toxin *E*. *coli* (LT), cholera toxin (CT), and their detoxified derivatives like LT-R129G/L211A or dmLT [1–4]. The latter is an advanced adjuvant candidate for both oral and parenteral vaccines [1]. When admixed with vaccine antigens, these protein adjuvants promote antigen-specific immune responses, including antibodies (e.g., IgG, IgA) and multipotent CD4 T-helper (Th)1/Th17/Th2 responses in both systemic and mucosal tissue compartments [1].

The LT and CT holotoxins have an AB_5_ structure composed of an enzymatic A-subunit non-covalently associated with a binding pentameric B-subunit. Binding and entry into host cells occurs through interactions of the B-subunit with gangliosides, particularly GM1, resulting in receptor-mediated endocytosis and retrograde transport to the golgi apparatus [5, 6]. The A-subunit is proteolytically cleaved by mucosal proteases (e.g., trypsin) at residue R192, creating an active A1 domain and an A2 peptide. Inside the golgi, the A1 domain is unraveled and transported through the sec61 pathway into the cytosol where it binds to cytosolic ADP-ribosylation factor (ARF). Together, A1 and ARF mediate ADP-ribosylation of Gsα, leading to irreversible adenylate cyclase activation, cAMP accumulation, and protein kinase A (PKA) activation, thereby inducing target protein phosphorylation [1]. CT, LT, dmLT and related mutant adjuvants activate APCs (e.g., monocytes, monocyte-derived dendritic cells [moDC], macrophages and DCs) in a process critical for the generation of post-vaccination responses, including upregulation of MHC-II, activation markers, and cytokine secretion [7–12]. Using murine bone marrow-derived DCs (BM-DCs), LT was shown to induce cytokine production via ERK MAPK signaling (e.g., IL-23 and IL-1α) or PKA signaling and NLRP3 inflammasome activation for IL-1β production [13]. Furthermore, mice deficient in IL-1 receptor (IL1R1-/-) could not produce antigen-specific Th17 responses after LT-adjuvanted vaccination. PBMCs or human monocytes stimulated with dmLT exhibited similar responses, including inflammasome gene expression and IL-1β cytokine secretion [14, 15]. The latter was required for antigen-specific IL-17A responses and was controlled by cAMP accumulation and PKA activation.

One problem with the holotoxin protein adjuvants and their AB_5_ mutants has been the potential for Bell’s palsy after intranasal administration [16, 17]. Thus, one option is to simply use the A1 domain of LT (LTA1) as an adjuvant to avoid the nasal toxicity observed in LT clinical studies. LTA1 does not bind to purified GM1 gangliosides *in vitro* but can still boost immunity to co-delivered antigens *in vivo*, including tetanus toxoid or ovalbumin [18]. In comparing the role of LT subunits on adjuvanticity, the quality (e.g., IgG1/IgG2 balance, mucosal IgA, and Th17 induction) of post-vaccination immune response was directed by the A-subunit with nasal delivery.

We previously observed upregulation of CD80 and CD86 costimulatory markers in BM-DCs treated with LTA or LTA1 treatment [18]. However, a thorough evaluation of LTA1 has never been performed in APCs. Thus, it is unclear if LTA1 activation of APCs would be altered compared to LT or dmLT, which could effect development of protective vaccine immunity. In the current investigation, we perform the first rigorous exploration of LTA1 activation of APCs using THP-1 cells that exhibit phenotypic plasticity, with selected validation in primary human monocytes. The established AB_5_ adjuvant dmLT acted as a relevant protein control to compare with LTA1. We also tested the hypothesis that LTA1 stimulates immunity through a mechanism that requires PKA activation but is GM1-independent, as expected.

## Results

### LTA1 and related dmLT adjuvant activate APCs, but do not promote macrophage-like phenotype in THP-1 cells

To investigate how LTA1 stimulates immunity, we performed a series of *in vitro* studies with THP-1 cells, a human monocyte cell line that exhibits plasticity to differentiate into macrophages or DC phenotypes [19, 20]. To understand how observed changes compared to an LT adjuvant containing an intact B subunit, we included a comparison to dmLT protein, which has never been evaluated in this model but has been reported to activate monocytes, BM-DCs, and *in vivo* DCs [11, 14, 15, 21]. Treatments were performed based on optimal vaccine doses reported in animal vaccine studies, including 10 μg LTA1 or 1 μg dmLT [22, 23]. Initially, we also included a PMA control which differentiates THP-1 cells into a macrophage phenotype (MΦ,[19]).

**Fig 1:**
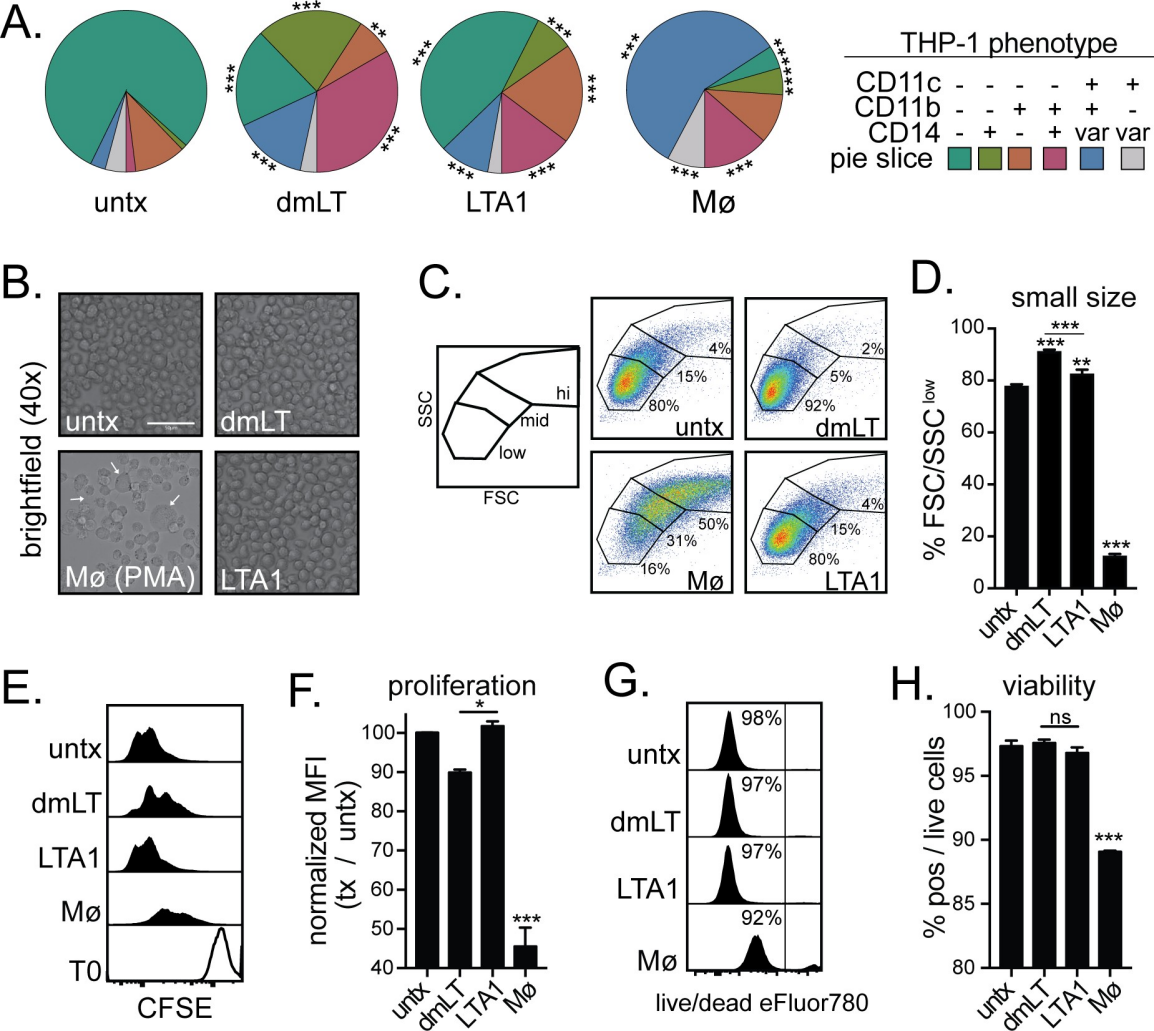
LTA1 and dmLT promote distinct changes in THP-1 cells unique from a macrophage phenotype. To evaluate changes to APCs, THP-1 cells were treated with media alone (untx) or containing 1 μg/ml dmLT, 10 μg/ml LTA1 or 10 ng/ml PMA (Mϕ). Cytometric samples and experiments were performed in triplicate. (A) Pie charts of THP-1 phenotypes determined after 48h treatment expressed as % cells expressing surface markers CD11c, CD11b, and/or CD14, including variable expression (var). (B) Representative images of treated cells by brightfield microscopy. Scale bar at 50 μm. Arrows indicate large adherent cells. (C) Representative FSC vs SSC dot blots with gating strategy indicated. (D) Percentage of cells with small size and low granularity based on FSC/SSC-low gating. (E) Representative histograms of CFSE at 0h (T0) or after 72h treatment. (F) Percentage of proliferating cells as reduced CFSE-expression relative to untreated. (G) Representative histograms viability dye used to label dead cells after 72h treatment. (H) Percentage of viable cells. Significance indicated as *P ≤ 0.05, **P ≤ 0.01, ***P ≤ 0.001 for two-way ANOVA with Bonferroni post-test for all groups (in A, with significance from untreated shown) or ANOVA with Bonferroni post-test for all groups compared to untx and as shown (in D, F, H). Bars at mean+SEM.

First we analyzed phenotypical changes of treated THP-1 cells. LTA1 and dmLT both significantly altered cell phenotypes from untreated, shifting from subpopulations lacking CD11c, CD11b and CD14 (teal green) to single, double or triple positive cells (olive green, orange, pink, blue populations; Fig 1A). Minor differences were seen between LTA1 and dmLT but did include higher expression of triple negative and CD11b single positive subpopulations with LTA1 treatment (teal green, orange) or CD14+ populations (olive green, pink) and CD11c, CD11b+, CD14-variable population (blue) with dmLT treatment. Although upregulation of these specific markers is associated with THP-1 differentiation into macrophages [20], we observed differences in comparison with MΦ culture conditions, which induced primarily a CD11c+, CD11b+, and CD14-variable population significantly larger than all other treatment conditions (blue).

We next analyzed THP-1 cells for other hallmarks of macrophage differentiation, such as increased plastic adherence, size or decreased cell viability. Plastic adherence and larger cells were observed only with MΦ treatment conditions (Fig 1B and 1C). In contrast, dmLT and LTA1 cells remained in suspension and actually became slightly smaller than untreated THP-1 cells (Fig 1C and 1D). Similarly, only MΦ treatment conditions significantly reduced cellular proliferation and viability (Fig 1E–1H). Although, a slight decrease in proliferation was observed with dmLT-treatment compared with LTA1 treated THP-1 cells, this decrease was not significantly lower than untreated cells. In conclusion, neither dmLT nor LTA1 caused THP-1 cells to differentiate into a MΦ phenotype.

### LTA1 and related dmLT adjuvant activate APCs, promoting a unique DC-like phenotype in THP-1 cells

**Fig 2:**
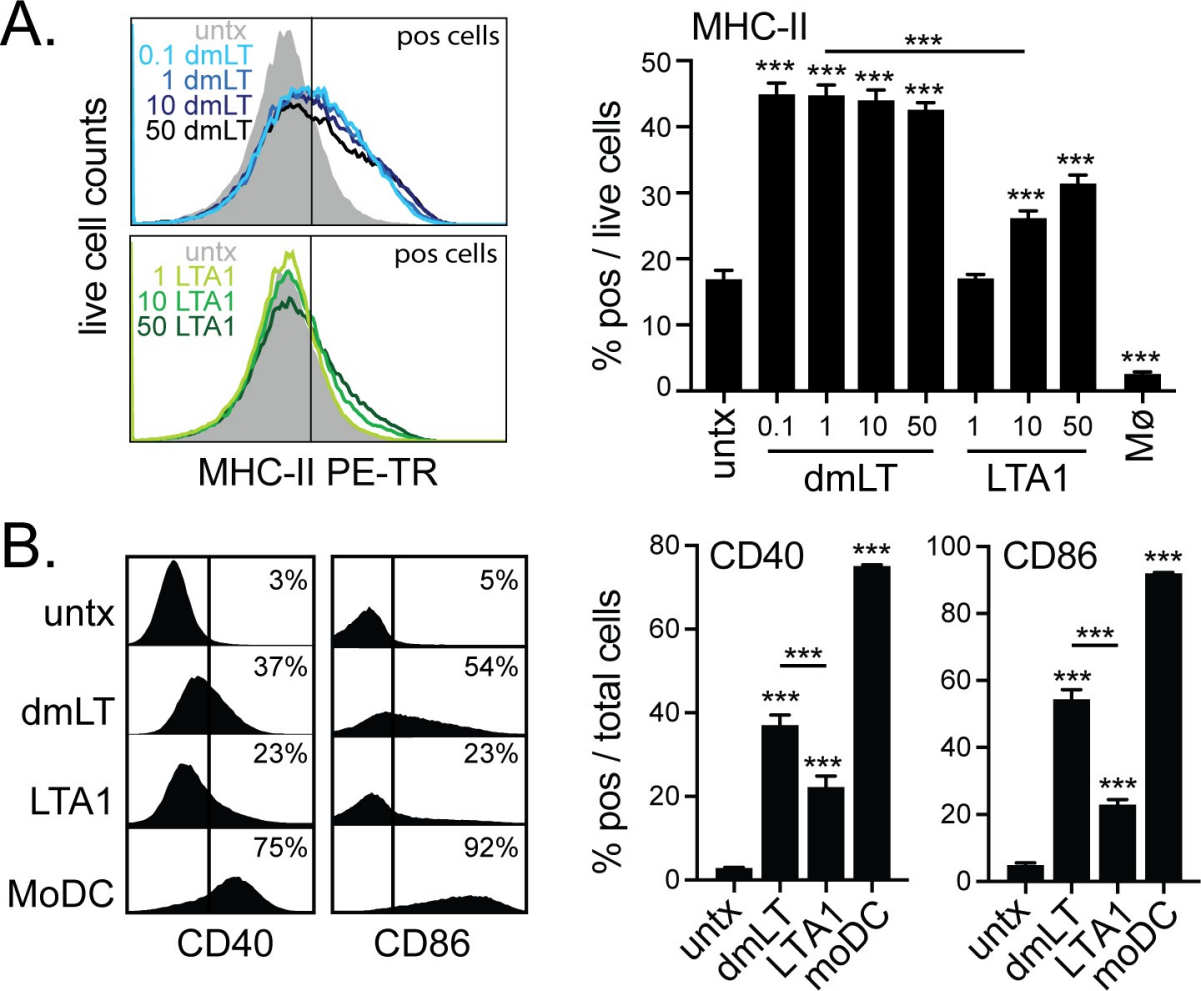
Treatment of THP-1 cells with LTA1 enhances surface markers indicative of DC-phenotype, but at lower levels than dmLT or moDC culture conditions. (A) THP-1 cells were treated for 72h with media alone (untx) and indicated μg dose/ml of dmLT, LTA1, or 10 ng/ml PMA (Mϕ). Representative histograms of MHC-II (HLA-DR) and % HLA-DR+ cells gated on live cells. (B) THP-1 cells were treated with media alone (untx), 1 μg/ml dmLT, 10μg/ml LTA1 or a moDC-inducing cocktail (ionomycin, IL-4, GM-CSF, TNF-α). Representative histograms of costimulatory molecules CD40 and CD86 and % positive cells gated on total cells after 72h treatment. Cytometric experiments performed with at least triplicate samples. Significance tested by ANOVA with Bonferroni post-test for all groups compared to untx and as indicated (*P ≤ 0.05, **P ≤ 0.01, ***P ≤ 0.001). Bars at mean+SEM.

A hallmark of DC differentiation by monocytes or THP-1 cells is upregulation of surface markers for antigen presentation including MHC-II, CD40, CD80, CD83, and CD86 as well as with cytokine secretion profiles linked to expansion of specific CD4 Th lineages (e.g., Th1, Th17, Th2, etc) [24–26]. To investigate if DC differentiation was occurring, we examined treated THP-1 cells for changes in these surface markers. LTA1 or dmLT treatment significantly enhanced expression of MHC-II (HLA-DR) unlike MΦ treatment conditions (Fig 2A and 2B). Using 0.1–50 μg/ml treatment doses, we found that HLA-DR expression was dose-independent with dmLT, but dose-dependent with LTA1. In addition, at highest dose tested, LTA1 treatment did not induced comparable levels of MHC-II as dmLT. Next we examined other markers of co-stimulation in comparison with cells treated to induce a monocyte-derived DC (moDC) phenotype using ionomycin and recombinant cytokines (IL-4, GM-CSF, TNF-α) [25]. LTA1 or dmLT treatment significantly enhanced expression of CD40 and CD86, but not CD80, CD83, or CD209 (Fig 2B and S1A Fig). Once again dmLT was significantly more potent than LTA1 at doses tested (despite differences in molar concentrations, e.g. 1 dmLT AB5 molecule to 4.07 molecules of LTA1 molecules at the same μg concentration), though not to the same level as moDC. In addition, neither dmLT nor LTA1 altered the response of THP-1 cells to LPS stimulation, in contrast to moDC (S1B–S1D Fig).

**Fig 3:**
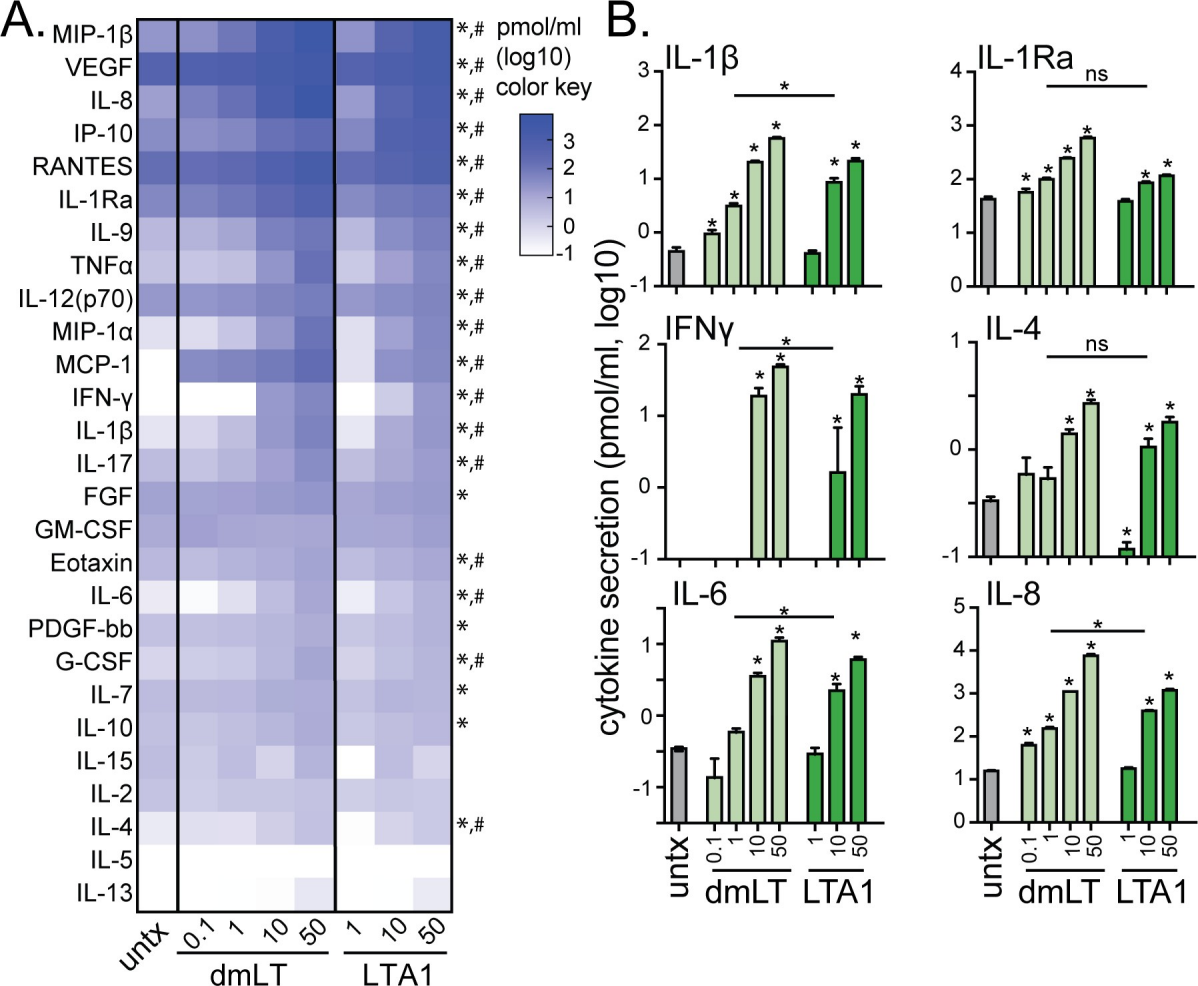
Treatment of THP-1 cells with LTA1 and dmLT induces comparable secretion of cytokines, including IL-1β, IFNγ, IL-4, and chemokines. To evaluate changes to APCs, THP-1 cells were treated with media alone (untx) or with dmLT or LTA1 in μg dose/ml indicated. Cytokine analyses were performed with triplicate samples. (A) Heatmap of secreted cytokines after 24h culture detected by Bio-plex. (B) Graphs of selected cytokines from A. Significance tested by ANOVA with Bonferroni post-test for all groups compared to untx and 1 μg dmLT to 10 μg LTA1 (indicated in A as * for dmLT or # for LTA1 both 10 and 50 μg/ml significantly higher than untx, or in B *P ≤ 0.05 for all comparisons). Bars at mean+SEM.

LTA1 and dmLT also stimulated cytokine secretion in treated THP-1 cells, with enhanced detection of INFγ, IL-12 (Th1-promoting), IL-4 (Th2-promoting), IL-1β and IL-6 (Th17-promoting) cytokines as well as inflammatory cytokines and chemokines involved in immune cell migration and activation (IL-8, IL-17, IP-10, G-CSF, MIP-1β, MCP-1, RANTES, TNFα, and VEGF, Fig 3 and S2 Fig). IL-1 receptor antagonist (IL-1Ra) was upregulated with treatment, and appeared to track similar to IL-1β secretion. In contrast to surface marker expression described above (Fig 2), cytokine expression was consistently similar between LTA1 and dmLT as well as dose-dependent. Thus, 10 μg LTA1 routinely resulted in higher levels of detected cytokines than 1 μg dmLT, but was fairly equivalent to 10 μg dmLT. These results indicate that dmLT and LTA1 induce a unique DC-like phenotype in THP-1 cells. They also indicate the magnitude of MHC-II, CD86, and CD40 expression is differentially controlled between LTA1 and dmLT from cytokine secretion, as the former was more robust with dmLT treatment while cytokine secretion was similar between LTA1 and dmLT.

### LTA1 and dmLT adjuvants activate IL-1β secretion through caspase-1 and inflammasome activation

**Fig 4:**
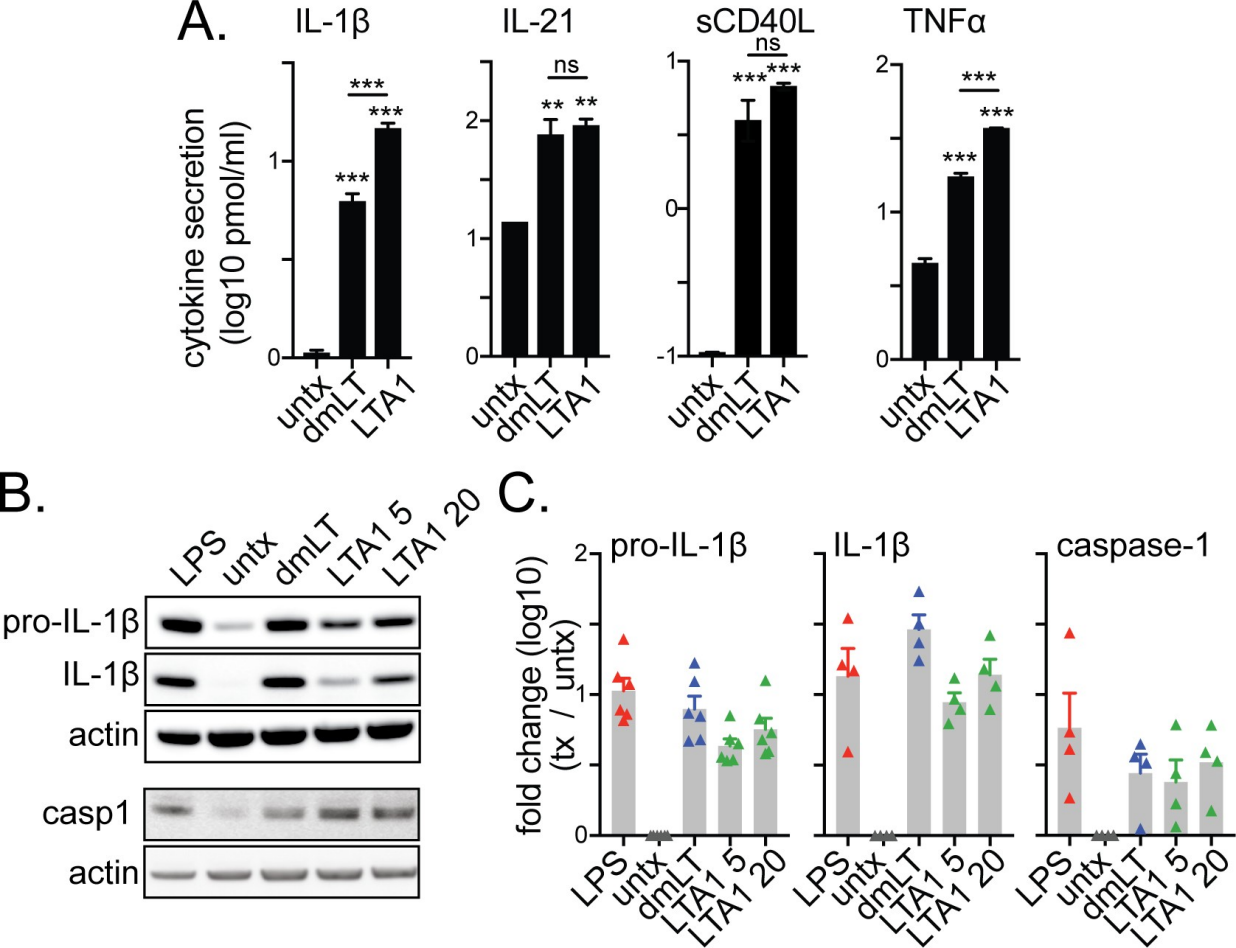
LTA1 and dmLT activate the inflammasome in APCs, including expression of caspase-1, pro-IL-1β, and cleaved IL-1β. (A) Secreted cytokines detected in 24h culture supernatants from THP-1 cells (0.8x10^6^/0.5ml) treated with media alone (untx) or with 1 ug/ml dmLT or 10 ug/ml LTA1. Signifiance tested by ANOVA with Bonferroni post-test for all groups compared to untx and as indicated (**P ≤ 0.01, ***P ≤ 0.001). (B) THP-1 cells were incubated with PMA for 12h and then treated with media alone (untx) or containing positive control 0.5 μg/ml LPS, 1 μg/ml dmLT, 5 or 20 μg/ml LTA1 for 12h. Representative Western blot images of cell lysates stained with anti- pro-IL-1β (band at 31kD), cleaved IL-1β (17kD), actin (42kD), and cleaved caspase-1 (Casp1, 20kD) antibodies. (C) Fold change from untx using relative intensity of protein bands normalized to actin control compiled from 4–6 separate experiments. Bars at mean+SEM.

Activation of the NLRP3 inflammasome leading to caspase-1 activation and IL-1β secretion has been highlighted as an important mechanism of dmLT and LT adjuvanticity [13–15]. To confirm that this is also occuring with LTA1 treatment, we performed additional testing. We cultured THP-1 cells at higher cell concentrations (0.8x10^6^/0.5ml) than normally recommended prior to treatment with 1 μg dmLT or 10 μg LTA1, rationalizing that any macrophage differentiation (in untreated cells) would be minimal after 24h while cytokine detection would be more easily discerned. Cells stimulated this way exhibited significant expression of IL-1β and TNFα as seen at lower cell concentrations (Fig 4A, S2 Fig and Fig 3) as well as germinal center promoting IL-21 and TNF family member soluble CD40L.

Next, we directly assessed inflammasome activation. Inflammasome activation occurs through a NFκB priming signal to initiate transcription of NLRP3 protein and a second signal that activates NLRP3 through oligomerization causing recruitment and autocatalysis of pro-caspase-1 to its active form (e.g., caspase-1) that ultimately cleaves IL-1β from its pro-form [27]. To study this, we first provided a priming signal by treating cells for 12h with PMA. Then we added LTA1 (5 or 20 μg/ml) or 1 μg/ml dmLT for an additional 12h as a potential secondary signal. LPS (0.5 μg/ml) served as a positive secondary signal control. Similar to LPS, both dmLT and LTA1 enhanced expression of protein bands for pro-IL-1β, cleaved IL-1β, and caspase-1 in cell lysates, which was repeated in multiple independent experiments (Fig 4B and 4C and S3 Fig).

### LTA1 activation of APCs is GM1-independent, in contrast to dmLT

**Fig 5:**
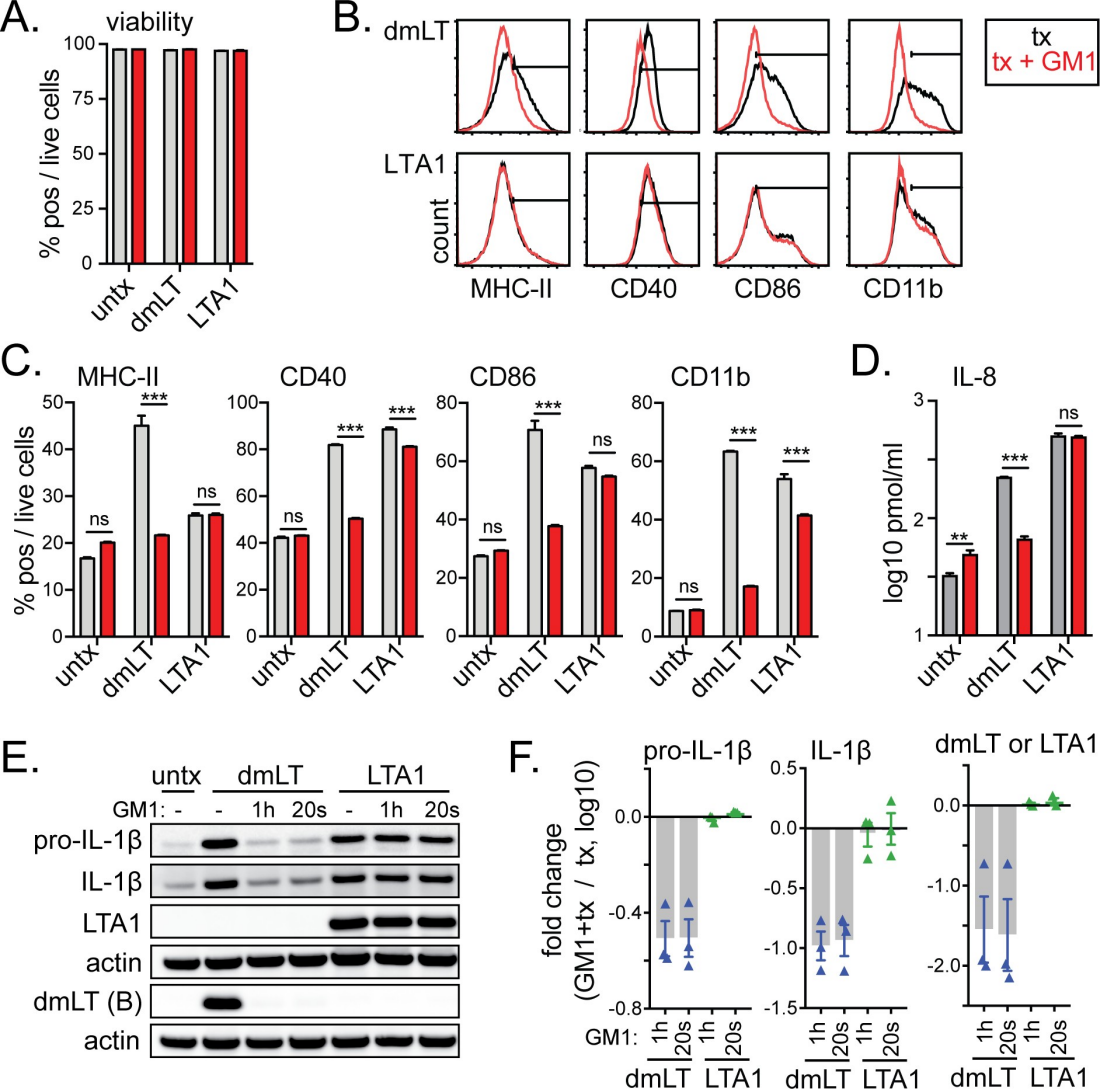
Unlike dmLT, LTA1 activation of APCs is GM1-independent. THP-1 cells were treated with media alone (untx), dmLT or LTA1 per 1x10^6^/ml cells (black lines or grey bars), or treatments pre-incubated for 15 min with 50μg/mL GM1 (red lines or bars). Cytometric samples and experiments performed in triplicate, cytokine analyses performed with triplicate samples. (A) Percentage of viable cells after 48h culture in media alone (untx) or with 1 μg/ml dmLT or 10 μg/ml LTA1. (B) Representative histograms of activation markers MHC-II (HLA-DR), CD40, CD86, or CD11b after 48h culture with positive gates shown. (C) Percentage surface marker positive cells gated on live cells. (D) Secreted IL-8 detected in 24h culture supernatants by Bio-plex. (E) Representative Western blot images for indicated protein bands using lysates of THP-1 cells incubated with PMA for 12h, then 50μg/ml GM1 for 1h or 20sec, then 1μg/ml dmLT or 20 μg/ml LTA1 for 12h as indicated. (F) Fold change from GM1+treatment using relative intensity of protein bands normalized to treatment compiled from 3 separate experiments. Significance tested by two-way ANOVA with Bonferroni post-test between selected pairs as indicated (**P ≤ 0.01, ***P ≤ 0.001). Bars at mean+SEM.

GM1 ganglioside binding by AB_5_ adjuvant proteins has been linked to neurologic toxicity and skin induration [23, 28, 29]; thus, an important aspect of the LTA1 protein should be lack of GM1 binding and internalization. LTA1 does not bind to recombinant GM1 by ELISA [18], but whether this is also true for immune cells has not been investigated. To determine whether GM1 binding is dispensable for LTA1-stimulated immunity we repeated select APC experiments in the presence of purified GM1. Once again, dmLT served as a control. Addition of GM1 to THP-1 cells did not alter cell viability with any treatment (Fig 5A). Incubation of LTA1 with GM1 prior to cell treatment did not alter MHC-II or CD86 expression, although some minor decreases in CD40 and CD11b were observed depending upon the experiment (Fig 5B and 5C). In contrast, incubation with GM1 prior to cell treatment completely ablated the ability of dmLT to upregulate any surface marker even with 3 days treatment. Similarly, detection of secreted IL-8 or expression of intracellular pro-IL-1β and cleaved IL-1β were not impaired for LTA1 when pre-incubated with GM1, but were for dmLT (Fig 5D–5F, S3 Fig). We did notice a slight, but not consistent reduction in LTA1 protein entry with the 20 μg/ml dose treatment pre-incubated with GM1 (S3 and S4 Figs), but this was not observed when GM1 was added directly to cell cultures for 1h or 20s prior to treatments (Fig 5E and 5F); whereas dmLT entry detected by B-subunit bands was inhibited in both experimental conditions. Thus, entry and activation of APCs by LTA1 protein is independent of GM1 binding, whereas dmLT’s ability to enter and activate cells can be blocked with exogenous GM1.

### Activation of APCs by LTA1 and dmLT does not require PKA activity

**Fig 6:**
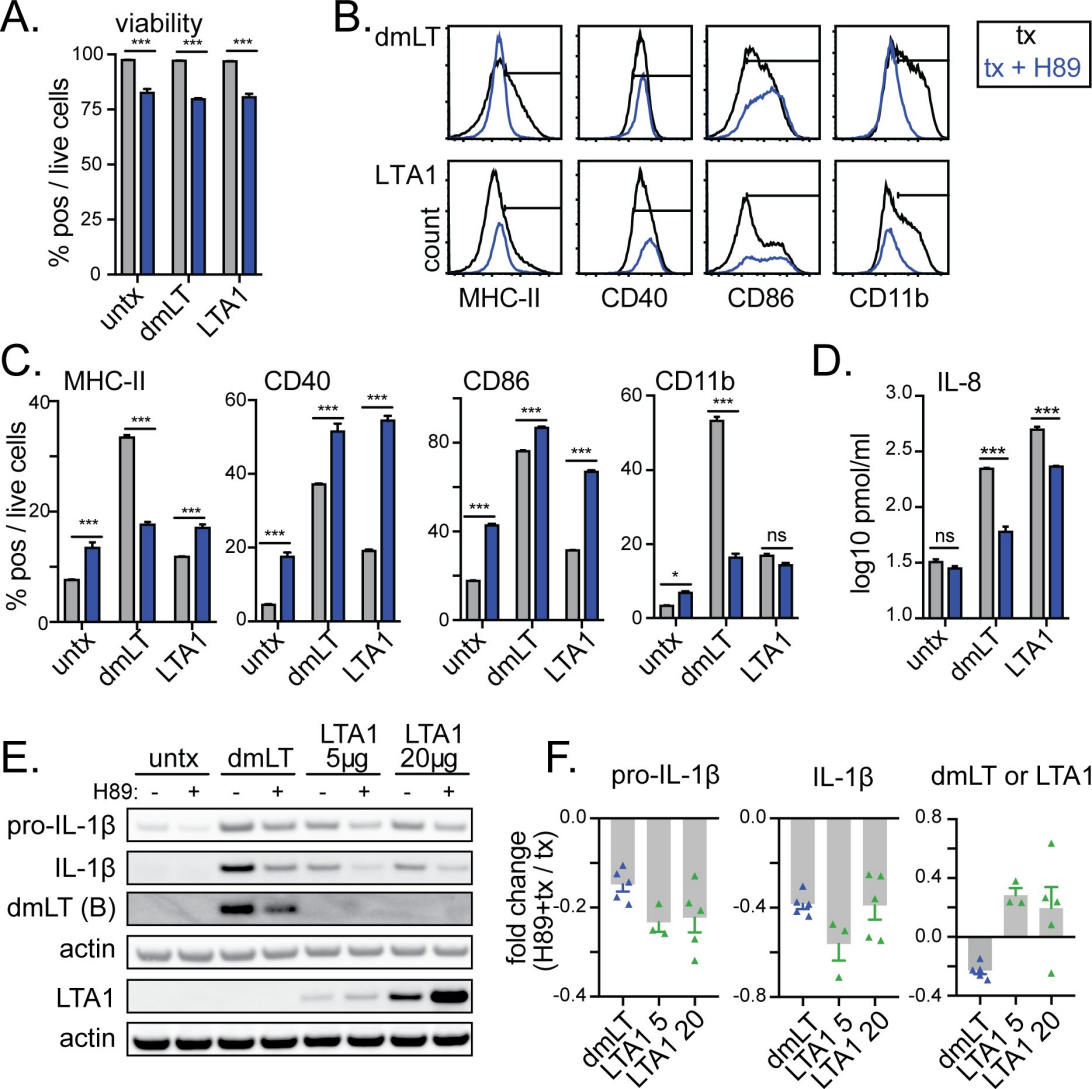
LTA1 and dmLT activation of APCs is partially prevented by PKA-inhibitor H89. THP-1 cells were treated with media alone (untx), 1 μg/ml dmLT or 10 μg/ml LTA1 (unless otherwise indicated) treatments per 1x10^6^/ml cells (black lines or grey bars) or pre-incubated for 1h with 20 μM H89 prior to treatments (blue lines or bars). Cytometric samples and experiments performed in triplicate, cytokine analyses performed with triplicate samples. (A) Percentage of viable cells after 48h culture. (B) Representative histograms of activation markers MHC-II (HLA-DR), CD40, CD86, or CD11b after 48h culture with positive gates shown. (C) Percentage surface marker positive cells gated on live cells. (D) Secreted IL-8 detected in 24h culture supernatants. (E) Representative Western blots images for indicated protein bands using lysates of THP-1 cells incubated with PMA for 12h and then media or H89 for 1 h prior to 1 μg/ml dmLT or 5–20 μg/ml LTA1 treatments as indicated. (F) Fold change from H89 + treatment using relative intensity of protein bands normalized to actin compiled from 3–5 separate experiments. Significance tested by two-way ANOVA with Bonferroni post-test between selected pairs as indicated (*P ≤ 0.05, ***P ≤ 0.001). Bars at mean+SEM.

Next, we evaluated whether responses to LTA1 were PKA-dependent, as previously reported for dmLT and LT, using the pharmacological inhibitor H89 [13–15]. Addition of H89 to THP-1 cells significantly reduced cell viability from ~97% to ~80%, but this reduction was consistent across treatment groups (Fig 6A). Prior treatment of cells with H89 did not significantly reduce expression of CD40, CD86, or CD11b, except for CD11b expression with dmLT (Fig 6B and 6C). Instead CD40 and CD86 were significantly increased in untreated, dmLT, and LTA1 treatment groups. MHC-II expression was inhibited with H89 in dmLT but not consistently in LTA1 treated groups. In contrast, H89 pre-treatment reduced IL-8 cytokine secretion and inflammasome activation detected as pro- and cleaved IL-1β bands by Western blot by both LTA1 and dmLT (Fig 6D–6F). Interestingly, H89 appeared to have the opposite effect on protein entry, with slight inhibition of dmLT band detection but slight increases in LTA1 band detection (Fig 6F).

**Fig 7:**
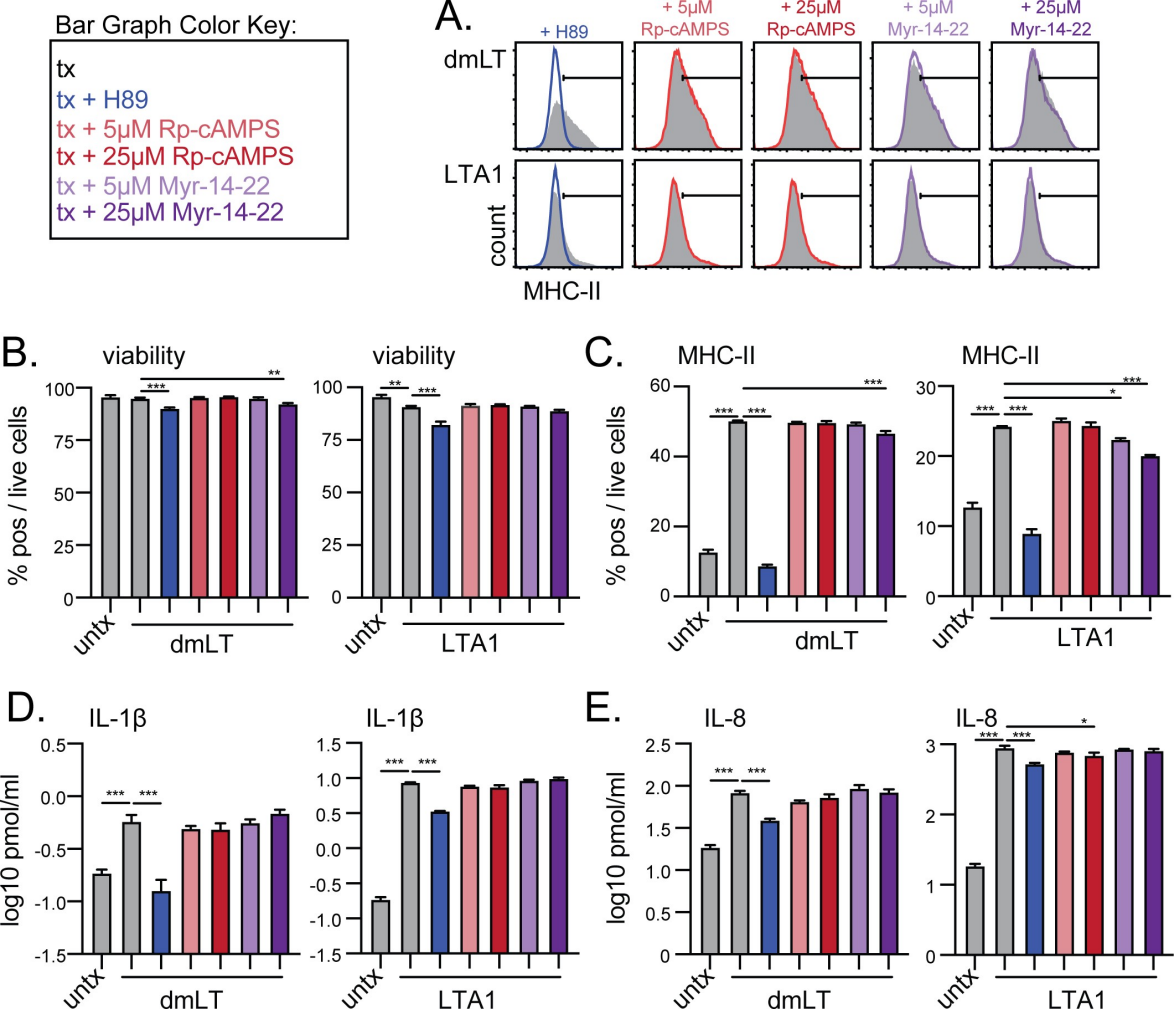
LTA1 and dmLT actiation of APCs is not prevented by PKA-specific inhibitors Myr-14-22 or *R*_p_-cAMPS. THP-1 cells were treated with media alone (untx), 1 μg/ml dmLT or 10 μg/ml LTA1 treatments per 1x10^6^/ml cells (grey bars or histograms) or pre-incubated for 1h with 20 μM H89, 5 or 25 μM Myr-14-22, or 5 or 25 μM *R*_p_-cAMPS prior to treatments (blue, red, and purple lines or bars). Cytometric samples and experiments performed in triplicate, cytokine analyses performed with triplicate samples. (A) Representative histograms of activation markers MHC-II (HLA-DR), after 24h culture with positive gates shown. (B) Percentage of viable cells after 24h culture. (C) Percentage MHC-II (HLA-DR) positive cells gated on live cells. (D) Secreted IL-1β detected in 24h culture supernatants. (E) Secreted IL-8 detected in 24h culture supernatants. Significance tested by ANOVA with Dunnett’s post-test for all compared to control dmLT or LTA1 treatment (*P ≤ 0.05, **P ≤ 0.01, ***P ≤ 0.001). Bars at mean+SEM.

While our results above agree with previous studies using H89 and LT proteins [13–15], H89 has been reported to have non-PKA specific pharmacologic effects [30, 31]. Thus, H89 cannot be used as definitive evidence for PKA-dependent effects, and so we next attempted to verify our results by using the specific PKA inhibitors Myr-14-22 or *R*_p_-cAMPS ([30], Fig 7A–7E). Addition of H89 or 25μM *R*_p_-cAMPS to THP-1 cells significantly reduced cell viability, but this reduction was minor and cell viability remained >80% (Fig 7B). While very minor changes were observed in MHC-II expression with pre-treatment with high doses of *R*_p_-cAMPS, only H89 fully inhibited or prevented the upregulation of MHC-II expression in dmLT or LTA1-stimulated cells to levels of untreated cells (Fig 7A and 7C). Similarly, only H89 pre-treatment reduced IL-1β and IL-8 cytokine secretion from dmLT or LTA1 (Fig 7D and 7E). These results indicate PKA-signaling is not the primary signaling pathway regulating MHC-II expression, inflammasome activation and cytokine secretion in APCs with LTA1 and dmLT proteins. Previous reports using H89 reported otherwise [13–15] were likely identifying a non-PKA specific effect of H89.

### Activation of APCs by LTA1 and dmLT is partially mimicked by dibutyryl cAMP or forskolin

**Fig 8:**
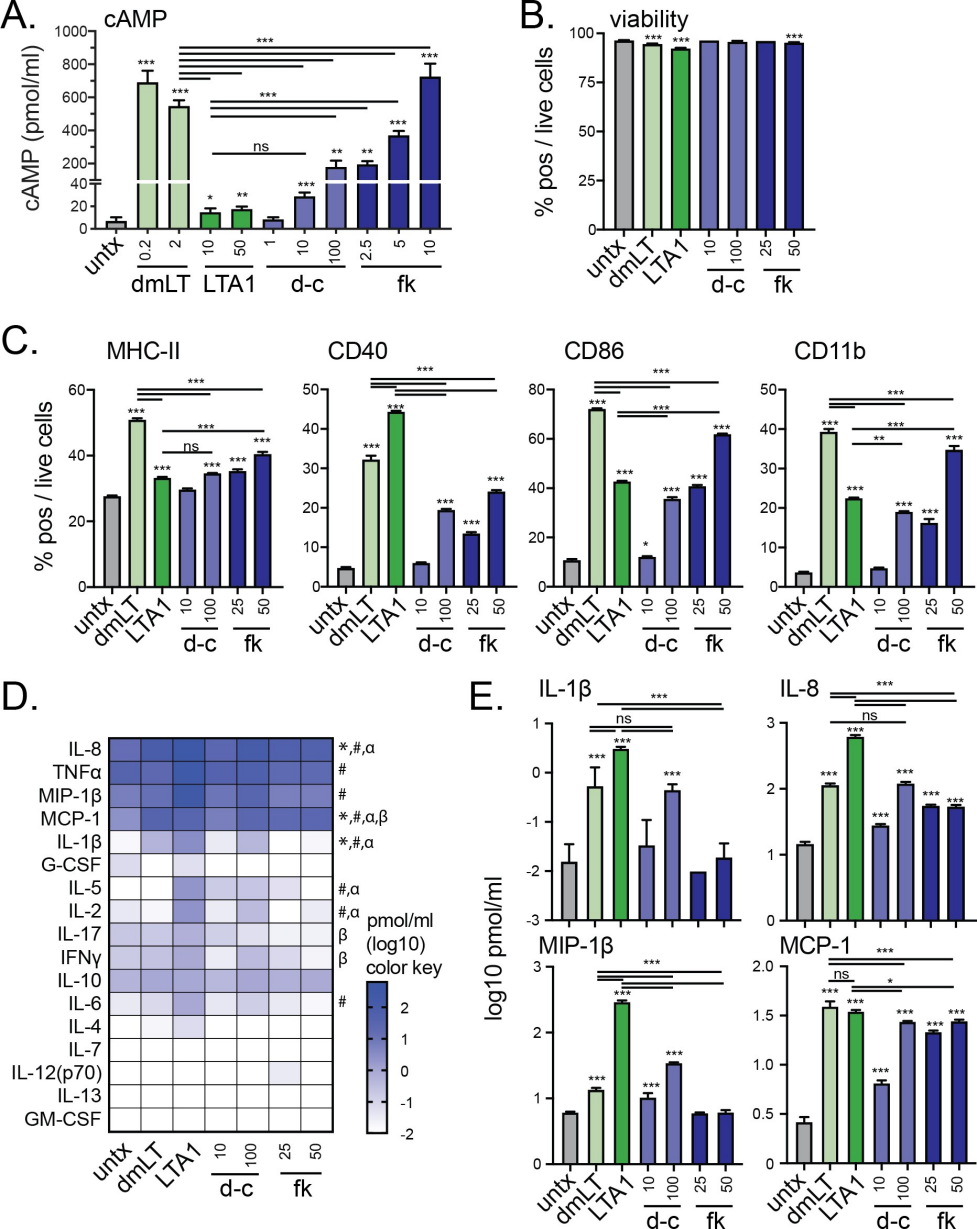
LTA1 and dmLT activation of APCs is partially recapitulated with dibutyryl cAMP or forskolin. THP-1 cells were treated with media alone (untx), 0.2–2 μg/ml dmLT, 10–50 μg/ml LTA1, 1–100 μΜ dibutyryl cAMP (d-c) or 2.5–50 μM forskolin (fk) treatments per 1x10^6^/ml cells. Cytometric samples and experiments performed in triplicate, cytokine analyses performed with triplicate samples. (A) Intracellular cAMP detection after 3h treatment in the presence of phosphodiesterase inhibitor. (B) Percentage viable cells gated on live cells after 72h treatment. (C) Percentage MHC-II (HLA-DR), CD40, CD86, or CD11b positive cells gated on live cells after 72h treatment (2 μg/ml dmLT, 10 μg/ml LTA1, or as indicated). (D) Heatmap of secreted cytokines indicated detected in 24h treatment (2 μg/ml dmLT, 10 μg/ml LTA1, or as indicated). (E) Graphs of selected secreted cytokines detected in 24h culture supernatants. Significance tested by with ANOVA with Bonferroni post-test to untreated or between selected pairs as indicated (*P ≤ 0.05, **P ≤ 0.01, ***P ≤ 0.001 in A-C,E) or two-way ANOVA with Bonferroni post-test for untx with significance *P* ≤ 0.05 indicated compared to dmLT (*), LTA1(^#^), d-c (^α^), or fk (^β^) in D. Bars at mean+SEM. n.s. = not significant.

The effect of LT proteins on APCs has frequently been reported to be mimicked by the cell permeable cAMP analog dibutyryl cAMP (d-cAMP) or the plant-derived pharmacological compound forskolin, that activates adenylate cyclase for cAMP production [7, 8, 14]. To compare the effects of LTA1 and dmLT in THP-1 cells compared with d-cAMP and forskolin we evaluated cAMP levels and changes to surface activation markers and secreted cytokines (Fig 8). cAMP levels were significantly altered by dmLT, LTA1, d-cAMP or forskolin treatments compared to untreated cells; however, significant difference were observed between dmLT and LTA1 treatments and d-cAMP and forskolin (Fig 8A). Notably, LTA1 induced low levels of cAMP that were comparable to 10 μM d-cAMP, while dmLT induced higher levels that were comparable to higher a dose of forskolin between 5–10 μM. Addition of similar treatments had some effects on THP-1 cell viability, but this reduction was minor and cell viability remained >92% (Fig 8B). Significant changes in MHC-II, CD40, CD86, and CD11b expression were observed with all treatments compared to untreated cells but with slightly different patterns than observed with cAMP induction (Fig 8C). This included that surface marker changes were much higher with dmLT even when compared with 25 or 50 μM forskolin and that CD40 expression with dmLT and LTA1 was higher than any d-cAMP or forskolin treatment. Similarly, while significant cytokine secretion was observed with all treatments, these were almost always much higher with LTA1 and dmLT treatments than with d-cAMP or forskolin (Fig 8D and 8E). These results indicate similarities in cAMP analogs/stimulating compounds and LTA1 and dmLT; however, as with our PKA experiments (Fig 7) differences in magnitude of these responses may be due to as yet undefined signaling pathways occurring with LTA1 and dmLT proteins and not simply level of cAMP induction.

### LTA1 stimulates activation of primary human monocytes, including IL-1β secretion

**Fig 9:**
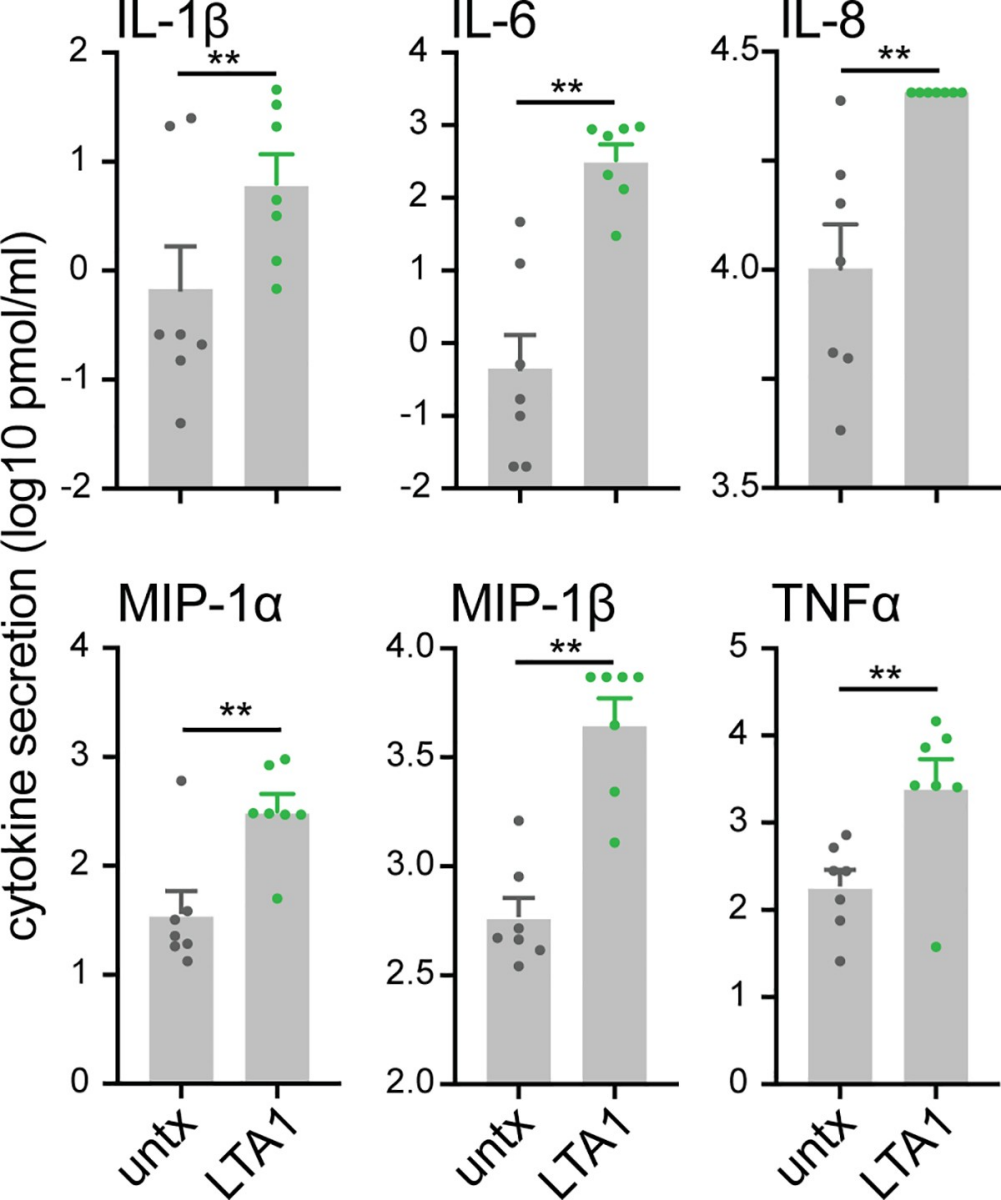
LTA1 stimulates activation of primary human monocytes, including secretion of cytokines. Human monocytes were purified from commercially purchased buffy coats from 7 donors and incubated for 3 days untreated (untx) or with 10 ug/ml LTA1. Secreted cytokines detected in 24h culture as log10 pg/ml. Samples above the interpolated range for MIP-1β and IL-8 were replaced with 0.01 over the highest interpolated value for that cytokine (3.87 or 4.41 respectively). Significance tested by paired two-tailed t-test (**P ≤ 0.01, ***P ≤ 0.001). Bars at mean+SEM.

Lastly, in order to confirm that these results have relevance to primary cells, we evaluate stimulation of monocytes from human donors. CD14+ monocytes from 7 donors were incubated for 3 days with or without LTA1 and evaluated for cytokine secretion. These primary human monocytes secreted IL-1β, IL-6, MIP-1α, MIP-1β, TNFα, and IL-8 which significantly (except for IL-8) increased after LTA1 treatment compared with untreated controls (Fig 9). Thus, LTA1 stimulates primary human cells similar to THP-1 cells.

## Discussion

LTA1 is being investigated as an alternative LT adjuvant protein and has recently been shown to safely improve vaccine outcomes intranasally in mice with flu vaccination or intradermally with enterotoxigenic *E*. *coli* vaccination [23, 29]. Here, we report the first detailed examination on activation of APCs by LTA1 protein. Using the THP-1 model, we confirmed stimulation and activation of a DC-like phenotype by LTA1. Surprisingly, these cellular effects and that related to dmLT did not require PKA activation, confirmed by use of specific inhibitors. However, we did find that LTA1 effects included inflammasome activation and were not GM1-dependent, thus partially confirming our hypothesis. In addition, while inflammasome activation has been inferred to be A-subunit dependent using AB_5_ mutant proteins, this is the first direct evidence that recombinant LTA1 protein can enter and activate the inflammasome through a cell entry mechanism distinct from AB_5_ mutant proteins. These results help to explain the adjuvanticity of recombinant LTA1 protein and have direct implications for vaccine development and use of LTA1 as a novel IN adjuvant.

In this study, we utilized the THP-1 model to study cellular activation. This cell line was derived from a patient with acute monocytic leukemia and exhibits the differentiation plasticity to stay monocyte-like or differentiate into macrophage or dendritic cell phenotypes [19, 20]. It is a common model to evaluate immune activation of myeloid cells, including the effects of adjuvant proteins [32], with the benefits of easy tissue-culture techniques and reduced variability compared with primary human cell culture or murine bone-marrow derived dendritic cells. This is the first use of this cell line to investigate LT-based proteins, although an older publication found IL-1β and IL-8 secretion in these cells after CT or heat-labile toxin II of *E*. *coli* (a similar enterotoxin to LT but with distinct binding subunit) [33]. In general, our results (Figs 1–3) were comparable with the effects that we and others have seen with upregulation of MHC-II, CD40 and CD86, as well as IL-1β, IL-6, IL-8, IL-10, IL-12p70 and TNFα secretion [8, 13, 18, 34–36]. Although, this is the first report of IL-21 and IL-1Ra cytokine release by LT enterotoxins and a thorough evaluation of the number of cytokine and chemokines induced after treatment with dmLT and LTA1 (Fig 3). IL-21 stimulates T follicular helper cells and plays an important role in germinal center maturation in lymphoid organs [37]. IL-1Ra is the receptor antagonist for IL-1 proteins, helping to control inflammation [38] and in our studies tracks with secretion of IL-1β. In addition, although we found that both dmLT and LTA1 resulted in a DC-like phenotype with upregulation of surface markers for antigen-presentation and cytokine secretion (Figs 1–3), these effects were distinctive from cytokine and ionomycin induced dendritic cell differentiation, including subsequent responses to LPS treatment (S1 Fig Supplemental). In addition, we observed less CD80 and CD83 expression after dmLT treatment than reported in other studies with dmLT or LT with primary monocytes, moDCs, B-cells and mouse BMDCs ([8, 18, 34, 35] and unpublished data). Thus, while THP-1 cells were an effective model for cellular activation with LT-based proteins, there are likely some limitations or cell-specific aspects to this model. To validate our findings, we were able to confirm that LTA1 activated IL-1β (and therefore the inflammasome), IL-6, IL-8, MIP-1α, MIP-1β, and TNFα secretion in both THP-1 cells and primary human monocytes (Figs 3, 9 and S2 Fig Supplemental). Thus, THP-1 cells were a relevant system to tease out the immunologic effects of LTA1 protein.

It has been recognized for some time that the complete immunologic effects of LT appear to require the A-subunit with some level of cAMP-induction. LT promotes *in vitro* dendritic cell activation, cytokine secretion, and Th17 cell induction through processes that can be approximated with cAMP analogs or other cAMP-inducing agents such as forskolin [7, 8]. Similarly, when the B-subunit or enzymatically-inactive LT mutants are compared with native LT, reduced or ablated adjuvant effects are observed *in vivo* or *in vitro* [8, 34, 35, 39, 40]. Our results indicate some unidentified signaling pathways are likely contributing to APC activation and not simply cAMP directed PKA activation of the inflammasome and cytokine secretion by LTA1 protein (Figs 6–8). Such an idea has not been characterized in the literature, as past studies have focused on PKA-regulation of Th17 responses in mice and human PBMCs by LT or AB_5_ mutant proteins; however these older studies relied on H89 for PKA inhibition [13–15], while it is clear that H89 can inhibit other cellular kinases, signaling molecules (e.g., calcium), cellular receptors and ion channels [30, 31]. Importantly, we also observed that patterns of cAMP induction with LTA1, dmLT, d-cAMP, and forskolin did not match that of APC activation, particularly cytokine secretion by LT proteins (Fig 8). What does seem to be clear with our results is that the A-subunit of LT directs inflammasome activation, IL-1β cleavage and overall levels of cytokine transcription and secretion; whereas the combination of intracellular signals provided by both the B-subunit and the A-subunit direct MHC-II and antigen presentation. Further studies into the precise signaling responses occurring with dmLT and LTA1 are warranted, including identifying alternative signaling pathways (including non-PKA targets of H89), transcription factors and consequences of GM1 binding/internalization.

Using exogenous GM1, we were able to confirm the differences between dmLT and LTA1. LTA1 protein has no specific binding target for cell entry (or at least is not engineered for such). This is in contrast to another well studied protein adjuvant formed from cholera toxin’s (CT) A-subunit genetically linked to two Ig-binding domains of staphylococcal protein A (CTA1-DD) that specifically target the molecule to B-cells [41, 42]. Furthermore, while LT and CT enterotoxins are similar, they have differential effects on the immune system; studies with CT and LT A/B_5_ hybrid toxins admixed in mouse vaccine studies indicate that IFNγ and IL-5 antigen-specific responses preferentially occur with LT and LTA/CTB but not with CT and CTA/LTB [43]. Similarly, PBMC stimulation with dmLT or CT result in comparable IL-17 secretion, but higher levels of cAMP and TNFα secretion with CT [14]. Thus, any effects attributed to CTA-DD studies, both in immune cell binding or adjuvanted vaccination outcomes cannot be assumed to be similar between LTA1 and CTA-DD. In our studies we were able to confirm LTA1 can activate THP-1 cells in the presence of purified exogenous GM1, in contrast to dmLT (Fig 5). While no entry mechanisms are confirmed at this point, we noticed a moderate increase in LTA1 protein with H89-inhibition in contrast to a moderate decrease in dmLT under the same conditions. Cellular kinases can regulate entry of viruses by altering receptor binding and entry [44]. Thus, we believe that LTA1 is likely entering through a non-receptor mediated mechanism like clathrin-mediated endocytosis and the macropinocytosis that has been suggested with other A-subunit studies in epithelial cell lines [45]. Whether these are the main entry mechanisms of LTA1 in antigen presenting cells warrants further study.

In conclusion, these results identify immune activation of THP-1 cells and human monocytes by LTA1 protein. These studies contribute to the understanding of LTA1 mechanisms of cellular activation and thereby ongoing development of LTA1 as an adjuvant protein for intranasal vaccination. In addition, by showing key similarities and differences between LTA1 and the AB_5_ adjuvant protein dmLT, we were able to contextualize our results in past research on LT proteins and also validate the THP-1 model as a useful system for continued exploration underlying mechanisms of these LT-based proteins.

## Materials and methods

### Vaccines and adjuvant proteins

LTA1 and dmLT were produced from *E*. *coli* clones expressing recombinant protein as previously described [3, 18]. Proteins were stored lyophilized and freshly resuspended prior to use (dmLT) or kept frozen at -20°C until use (LTA1). The endotoxin content of all proteins was <1 EU/mg.

### Thp-1 culture experiments

THP-1 cells (ATCC® TIB-202^™^, Manassas, VA) were passaged as recommended at ≤1x10^6^ cells/ml. Unless otherwise indicated, cells were treated in 24-well plates for 12–72 h with indicated μg/ml concentrations of dmLT, LTA1, or μM concentrations of purchased dibutyryl cyclic-AMP sodium salt or forskolin (Millipore Sigma), to yield 0.8-1x10^6^ cells/ml upon sample collection. MΦ differentiation was induced by treating THP-1 monocytes (5×10^5^ cells/ml) with 10 ng/mL phorbol 12-myristate 13-acetate (PMA, Sigma–Aldrich) for 48 h followed by 24 h incubation in fresh media. For moDC differentiation, THP-1 cells were harvested, washed and resuspended in serum-free culture medium with the following cytokines: 200 ng/ml rhIL-4, 100 ng/ml rhGM-CSF, 20 ng/ml rhTNF-α (eBiosciences) and 200 ng/ml ionomycin (Millipore Sigma). Cells were seeded at a concentration of 4×10^5^ cells/ml. Twenty-four hours later, 10% FBS was added and cells were cultured for 48 h. For LPS challenge experiments, after treatment cells were incubated in fresh media with or without 10 ng/ml LPS (Sigma, Ecoli 055:B5) for 24 h. For inhibition experiments, cells were either pre-incubated with 20 μM H89 (Millipore Sigma), 5 or 25 μM PKA inhibitor 14–22 Amide, Cell-Permeable, Myristoylated (PKI-(Myr-14-22), CalBiochem), 5 or 25 μM Rp-Diastereomer of adenosine-3′,5′-cyclic monophosphothioate (*R*_p_-cAMPS Millipore Sigma), or 50 μg/ml monosialoganglioside GM1 (GM1, Millipore Sigma) and followed by treatment with dmLT or LTA1 as indicated or GM1 was pre-incubated with dmLT or LTA1 in 50 μl for 15 min at 20°C and then added to the wells achieving a final concentration of 50 μg/ml GM1. Cultured cells were photographed using EVOS XL core microscope (Life Technologies, Carlsbad, CA, USA).

### Flow cytometry

For immunofluorescence staining, cells were stained using fixable viability dye eFluor 780 (ThermoFisher Sci., Waltham, MA) and antibodies to surface markers including: anti-human CD14 conjugated to eFlour450 (clone 61D3), CD11b FITC (ICRF44), CD86 PE-Cy7 (B7-2), CD40 eF450 (5C3) (ThermoFisher Sci.), CD11c PE-Cy7 (3.9) HLA-DR PE-Dazzle (L243) (BioLegend, San Diego, CA). For proliferation staining, THP-1 cells were stained with 1 uM CFSE (ThermoFisher Sci.), incubated for 10 min at 37°C, washed with warm complete media and resuspended 2x10^5^ cells/ml in fresh media. Next, cells were plated, treated and cultured for 72 h. Stained cells were fixed with 2% paraformaldehyde (PolySciences Inc., Warrington, PA). Cytometric analyses were performed on BD LSRFortessa and analyzed using FlowJo v10 software (Ashland, OR).

### Cytokine measurements

Cytokines were analyzed with thawed supernatants using human Bio-Plex Pro^™^ Human Cytokine 27-plex, Th17 Cytokine Panel or custom arrays with a Bio-plex 200 array reader (Bio-Rad, Hercules, CA). For human confirmation of THP1 results, some LTA1 treated samples were above the interpolatable range for MIP-1β and IL-8 respectively. These values were replaced with 0.01 over the highest interpolated value for that cytokine.

### Western blot analyses

Treated cells were washed, detached with scraping, pelleted, resuspended with Nupage SDS loading buffer (ThermoFisher Sci.), and lysed with three 10s pulses at 40% sonication power (Heat Systems Ultrasonics, Plainview, NY). Lysates were loaded into NuPage 12% or 4–12% Bis-Tris gel wells (ThermoFisher Sci.) for gel electrophoresis, then transferred to nitrocellulose membrane with iBlot and developed using Inflammasome Antibody Sampler Kit #32961T (CST Danvers, MA) or rabbit anti-LTA or LT-B as previously described [46]. Blots were imaged with Pierce^™^ ECL Western Blotting Substrate (ThermoFisher Sci.) and Amersham Imager 600 and quantified using ImageQuant LT (GE Healthcare, Pittsburgh, PA).

### cAMP detection

Intracellular cAMP levels were detected similar to previous reports [3, 18]. Briefly, 0.5x10^6^ cells/0.5ml THP-1 cells in 24-well plates were washed in serum free media and then pre-treated cultured with 50 μM rolipram (Sigma) a phosphodiesterase inhibitor for 1h in RPMI-1640 media containing 1% FBS, followed by a 3h treatment with various proteins or compounds. Intracellular cAMP levels were then detected with cyclic AMP Immunoassay kit (R&D Systems, Minneapolis, MN) following manufacturer’s instructions.

### Monocyte culture

CD14 positive monocytes were purified from buffy coats purchased from The Blood Center (New Orleans, LA), using EasySep Direct Human Monocyte Isolation Kit for immunomagnetic negative selection (STEMCELL Technologies). Cells were seeded at 2x10^6^ cells/ml in serum-free CTL test media, incubated for 3 days with or without 10ug/ml LTA1 and had supernatants were collected.

### Statistical analysis

Statistical analyses were performed using GraphPad Prism v.7.0 (La Jolla, CA). Cytokine levels were log10 transformed for normalcy and homogeneity of variance. Statistics were performed using paired two-tailed t-test for comparisons of two groups. For comparisons of more than two groups, one- or two-way ANOVA was used with Dunnett’s post-test for all compared to control or Bonferroni post-test otherwise. Pie charts were created using SPICE software [47]. Final Figs were prepared using Adobe Illustrator software (San Jose, CA).

## Supporting information

S1 Fig THP-1 cells treated with dmLT and LTA1 do not show complete overlap with moDC phenotype and cytokine secretion. To evaluate changes to APCs, THP-1 cells were treated with media alone (untx), with 1 μg/ml dmLT or 10μg/ml LTA1, or ionomycin and cytokine (IL-4, GM-CSF, TNF-α) to induce differentiation into dendritic cells (moDC). After treatment, cells were incubated in fresh media with or without 10ng/ml LPS for 24h. Cytometric samples and experiments were performed in triplicate; cytokine analyses were performed with triplicate samples. (A) Representative histograms for costimulatory molecules CD80, CD83, and CD209. (B) Representative CD40 vs CD80 dot plots from untx cells with or without LPS stimulation. (C) Mean+SEM positive cells gated for total cells. Significance tested by two-way ANOVA with Bonferroni post-test between selected pairs as indicated (*P ≤ 0.05, **P ≤ 0.01, ***P ≤ 0.001). Bars at mean+SEM.
(TIF)

S2 Fig Treatment of THP-1 cells with LTA1 and dmLT induces comparable secretion of cytokines. To evaluate changes to APCs, THP-1 cells were treated with media alone (untx) or with dmLT or LTA1 in μg doses/ml indicated or 10 ng/ml PMA (Mϕ). Cytokine analyses were performed with triplicate samples. Selected mean+SEM secreted cytokines after 24h culture detected by Human 27-plex Bioplex are shown. Significance tested by ANOVA with Bonferroni post-test for all groups compared to untx and as indicated (*P ≤ 0.05).
(TIF)

S3 Fig Uncropped Western blot images. Uncropped jpg ECL images of Western blots merged with brightfield images showing colorimetric standard SeeBlue Plus 2 and detection antibody indicated on top of image. Rectangle selections indicate cropped images used in Fig 4B (A), Fig 5E (B), S4A Fig Supplemental (C) and Fig 6E (D).
(TIF)

S4 Fig Unlike dmLT, LTA1 activation of the inflammasome is GM1-independent. THP-1 cells (0.5e6/ml) were incubated with PMA for 12 h then left untreated (untx) or stimulated for 12h with positive control 1 μg/ml LPS, 0.5 μg/ml dmLT, or 5–20 μg/ml LTA1 as indicated. Experiments performed at least in triplicate. In some cases, treatments were pre-incubated with GM1 for 15 min at 20C prior to cell treatments. (A) Representative Western blots images for indicated protein bands using lysates of THP-1 cells. (B) Fold change of GM1+treatment from treatment using relative intensity of protein bands normalized to actin compiled from 3 or more separate experiments. Bars at mean+SEM.
(TIF)

## References

[1] Clements JD, Norton EB. The Mucosal Vaccine Adjuvant LT(R192G/L211A) or dmLT. mSphere. 2018;3(4). 10.1128/mSphere.00215-18 30045966 PMC6060342

[2] Clements JD, Hartzog NM, Lyon FL. Adjuvant activity of *Escherichia coli* heat-labile enterotoxin and effect on the induction of oral tolerance in mice to unrelated protein antigens. Vaccine. 1988;6(3):269–77. Epub 1988/06/01. 10.1016/0264-410x(88)90223-x . 3048010

[3] Norton EB, Lawson LB, Freytag LC, Clements JD. Characterization of a mutant Escherichia coli heat-labile toxin, LT(R192G/L211A), as a safe and effective oral adjuvant. Clin Vaccine Immunol. 2011;18(4):546–51. Epub 2011/02/04. doi: CVI.00538-10 [pii] 10.1128/CVI.00538-10 . 21288994 PMC3122563

[4] Ogra PL, Faden H, Welliver RC. Vaccination strategies for mucosal immune responses. Clin Microbiol Rev. 2001;14(2):430–45. 10.1128/CMR.14.2.430-445.2001 11292646 PMC88982

[5] Moss J, Osborne JC Jr., Fishman PH, Nakaya S, Robertson DC. Escherichia coli heat-labile enterotoxin. Ganglioside specificity and ADP-ribosyltransferase activity. J Biol Chem. 1981;256(24):12861–5. . 6273411

[6] Mudrak B, Kuehn MJ. Heat-labile enterotoxin: beyond G(m1) binding. Toxins (Basel). 2010;2(6):1445–70. Epub 2010/06/01. 10.3390/toxins2061445 22069646 PMC3153253

[7] Anosova NG, Chabot S, Shreedhar V, Borawski JA, Dickinson BL, Neutra MR. Cholera toxin, *E*. *coli* heat-labile toxin, and non-toxic derivatives induce dendritic cell migration into the follicle-associated epithelium of Peyer's patches. Mucosal Immunol. 2008;1(1):59–67. Epub 2008/12/17. 10.1038/mi.2007.7 [pii] 19079161 PMC2614317

[8] Bagley KC, Abdelwahab SF, Tuskan RG, Fouts TR, Lewis GK. Cholera toxin and heat-labile enterotoxin activate human monocyte-derived dendritic cells and dominantly inhibit cytokine production through a cyclic AMP-dependent pathway. Infection and immunity. 2002;70(10):5533–9. Epub 2002/09/14. 10.1128/IAI.70.10.5533-5539.2002 12228279 PMC128358

[9] Fahlen-Yrlid L, Gustafsson T, Westlund J, Holmberg A, Strombeck A, Blomquist M, et al CD11c(high) dendritic cells are essential for activation of CD4+ T cells and generation of specific antibodies following mucosal immunization. J Immunol. 2009;183(8):5032–41. Epub 2009/09/30. doi: jimmunol.0803992 [pii] 10.4049/jimmunol.0803992 . 19786541

[10] Heine SJ, Diaz-McNair J, Andar AU, Drachenberg CB, van de Verg L, Walker R, et al Intradermal delivery of *Shigella* IpaB and IpaD type III secretion proteins: kinetics of cell recruitment and antigen uptake, mucosal and systemic immunity, and protection across serotypes. J Immunol. 2014;192(4):1630–40. 10.4049/jimmunol.1302743 24453241 PMC3998105

[11] Novotny LA, Clements JD, Bakaletz LO. Transcutaneous immunization as preventative and therapeutic regimens to protect against experimental otitis media due to nontypeable *Haemophilus influenzae*. Mucosal immunology. 2011;4(4):456–67. 10.1038/mi.2011.6 21326197 PMC3118858

[12] Ryan EJ, McNeela E, Pizza M, Rappuoli R, O'Neill L, Mills KH. Modulation of innate and acquired immune responses by Escherichia coli heat-labile toxin: distinct pro- and anti-inflammatory effects of the nontoxic AB complex and the enzyme activity. J Immunol. 2000;165(10):5750–9. Epub 2000/11/09. 10.4049/jimmunol.165.10.5750 . 11067933

[13] Brereton CF, Sutton CE, Ross PJ, Iwakura Y, Pizza M, Rappuoli R, et al *Escherichia coli* heat-labile enterotoxin promotes protective Th17 responses against infection by driving innate IL-1 and IL-23 production. J Immunol. 2011;186(10):5896–906. Epub 2011/04/15. doi: jimmunol.1003789 [pii] 10.4049/jimmunol.1003789 . 21490151

[14] Larena M, Holmgren J, Lebens M, Terrinoni M, Lundgren A. Cholera toxin, and the related nontoxic adjuvants mmCT and dmLT, promote human Th17 responses via cyclic AMP-protein kinase A and inflammasome-dependent IL-1 signaling. J Immunol. 2015;194(8):3829–39. 10.4049/jimmunol.1401633 . 25786687

[15] Leach S, Clements JD, Kaim J, Lundgren A. The adjuvant double mutant Escherichia coli heat labile toxin enhances IL-17A production in human T cells specific for bacterial vaccine antigens. PLoS One. 2012;7(12):e51718 10.1371/journal.pone.0051718 23284753 PMC3527457

[16] Lewis DJ, Huo Z, Barnett S, Kromann I, Giemza R, Galiza E, et al Transient facial nerve paralysis (Bell's palsy) following intranasal delivery of a genetically detoxified mutant of Escherichia coli heat labile toxin. PLoS One. 2009;4(9):e6999 10.1371/journal.pone.0006999 19756141 PMC2737308

[17] Mutsch M, Zhou W, Rhodes P, Bopp M, Chen RT, Linder T, et al Use of the inactivated intranasal influenza vaccine and the risk of Bell's palsy in Switzerland. N Engl J Med. 2004;350(9):896–903. 10.1056/NEJMoa030595 . 14985487

[18] Norton EB, Lawson LB, Mahdi Z, Freytag LC, Clements JD. The A subunit of Escherichia coli heat-labile enterotoxin functions as a mucosal adjuvant and promotes IgG2a, IgA, and Th17 responses to vaccine antigens. Infect Immun. 2012;80(7):2426–35. 10.1128/IAI.00181-12 22526674 PMC3416479

[19] Chanput W, Mes JJ, Wichers HJ. THP-1 cell line: an in vitro cell model for immune modulation approach. Int Immunopharmacol. 2014;23(1):37–45. Epub 2014/08/14. 10.1016/j.intimp.2014.08.002 25130606

[20] Mittar D, Paramban R, McIntyre C. Flow Cytometry and High-Content Imaging to Identify Markers of Monocyte-Macrophage Differentiation. 2011.

[21] Frederick DR, Goggins JA, Sabbagh LM, Freytag LC, Clements JD, McLachlan JB. Adjuvant selection regulates gut migration and phenotypic diversity of antigen-specific CD4(+) T cells following parenteral immunization. Mucosal immunology. 2017 10.1038/mi.2017.70 . 28792004 PMC6252260

[22] Norton EB, Bauer DL, Weldon WC, Oberste MS, Lawson LB, Clements JD. The novel adjuvant dmLT promotes dose sparing, mucosal immunity and longevity of antibody responses to the inactivated polio vaccine in a murine model. Vaccine. 2015;33(16):1909–15. 10.1016/j.vaccine.2015.02.069 . 25765967

[23] Valli E, Harriett AJ, Nowakowska MK, Baudier RL, Provosty WB, McSween Z, et al LTA1 is a safe, intranasal enterotoxin-based adjuvant that improves vaccine protection against influenza in young, old and B-cell-depleted (μMT) mice. Sci Rep. 2019;9(15128). 10.1038/s41598-019-51356-w PMC6805908 31641151

[24] Jakubzick CV, Randolph GJ, Henson PM. Monocyte differentiation and antigen-presenting functions. Nat Rev Immunol. 2017;17(6):349–62. Epub 2017/04/24. 10.1038/nri.2017.28 . 28436425

[25] Berges C, Naujokat C, Tinapp S, Wieczorek H, Hoh A, Sadeghi M, et al A cell line model for the differentiation of human dendritic cells. Biochem Biophys Res Commun. 2005;333(3):896–907. Epub 2005/06/21. 10.1016/j.bbrc.2005.05.171 . 15963458

[26] Zhu J, Yamane H, Paul WE. Differentiation of effector CD4 T cell populations (*). Annu Rev Immunol. 2010;28:445–89. Epub 2010/03/03. 10.1146/annurev-immunol-030409-101212 20192806 PMC3502616

[27] Jo EK, Kim JK, Shin DM, Sasakawa C. Molecular mechanisms regulating NLRP3 inflammasome activation. Cell Mol Immunol. 2016;13(2):148–59. Epub 2015/11/10. 10.1038/cmi.2015.95 26549800 PMC4786634

[28] van Ginkel FW, Jackson RJ, Yoshino N, Hagiwara Y, Metzger DJ, Connell TD, et al Enterotoxin-based mucosal adjuvants alter antigen trafficking and induce inflammatory responses in the nasal tract. Infect Immun. 2005;73(10):6892–902. Epub 2005/09/24. doi: 73/10/6892 [pii] 10.1128/IAI.73.10.6892-6902.2005 16177369 PMC1230900

[29] Maciel M Jr., Bauer D, Baudier RL, Bitoun J, Clements JD, Poole ST, et al Intradermal or Sublingual Delivery and Heat-Labile Enterotoxin Proteins Shape Immunologic Responses to a CFA/I Fimbria-Derived Subunit Antigen Vaccine against Enterotoxigenic Escherichia coli. Infect Immun. 2019;87(11). Epub 2019/08/21. 10.1128/IAI.00460-19 31427449 PMC6803349

[30] Murray AJ. Pharmacological PKA inhibition: all may not be what it seems. Sci Signal. 2008;1(22):re4 Epub 2008/06/05. 10.1126/scisignal.122re4 . 18523239

[31] Limbutara K, Kelleher A, Yang CR, Raghuram V, Knepper MA. Phosphorylation Changes in Response to Kinase Inhibitor H89 in PKA-Null Cells. Sci Rep. 2019;9(1):2814 Epub 2019/02/28. 10.1038/s41598-019-39116-2 30808967 PMC6391403

[32] Okemoto K, Kawasaki K, Hanada K, Miura M, Nishijima M. A potent adjuvant monophosphoryl lipid A triggers various immune responses, but not secretion of IL-1beta or activation of caspase-1. J Immunol. 2006;176(2):1203–8. Epub 2006/01/06. 10.4049/jimmunol.176.2.1203 . 16394010

[33] Hajishengallis G, Nawar H, Tapping RI, Russell MW, Connell TD. The Type II heat-labile enterotoxins LT-IIa and LT-IIb and their respective B pentamers differentially induce and regulate cytokine production in human monocytic cells. Infect Immun. 2004;72(11):6351–8. Epub 2004/10/27. 10.1128/IAI.72.11.6351-6358.2004 15501764 PMC523043

[34] Cheng E, Cardenas-Freytag L, Clements JD. The role of cAMP in mucosal adjuvanticity of Escherichia coli heat-labile enterotoxin (LT). Vaccine. 1999;18(1–2):38–49. Epub 1999/09/29. 10.1016/s0264-410x(99)00168-1 . 10501233

[35] Negri DR, Pinto D, Vendetti S, Patrizio M, Sanchez M, Riccomi A, et al Cholera toxin and Escherichia coli heat-labile enterotoxin, but not their nontoxic counterparts, improve the antigen-presenting cell function of human B lymphocytes. Infect Immun. 2009;77(5):1924–35. Epub 2009/02/19. doi: IAI.01559-08 [pii] 10.1128/IAI.01559-08 19223474 PMC2681738

[36] Read LT, Hahn RW, Thompson CC, Bauer DL, Norton EB, Clements JD. Simultaneous exposure to Escherichia coli heat-labile and heat-stable enterotoxins increases fluid secretion and alters cyclic nucleotide and cytokine production by intestinal epithelial cells. Infect Immun. 2014;82(12):5308–16. Epub 2014/10/08. 10.1128/IAI.02496-14 25287923 PMC4249298

[37] Vogelzang A, McGuire HM, Yu D, Sprent J, Mackay CR, King C. A fundamental role for interleukin-21 in the generation of T follicular helper cells. Immunity. 2008;29(1):127–37. Epub 2008/07/08. 10.1016/j.immuni.2008.06.001 . 18602282

[38] Arend WP, Guthridge CJ. Biological role of interleukin 1 receptor antagonist isoforms. Ann Rheum Dis. 2000;59 Suppl 1:i60–4. Epub 2000/10/29. 10.1136/ard.59.suppl_1.i60 11053091 PMC1766634

[39] Lobet Y, Cluff CW, Cieplak W Jr. Effect of site-directed mutagenic alterations on ADP-ribosyltransferase activity of the A subunit of *Escherichia coli* heat-labile enterotoxin. Infection and immunity. 1991;59(9):2870–9. Epub 1991/09/11. 1908825 10.1128/iai.59.9.2870-2879.1991 PMC258107

[40] Lycke N, Tsuji T, Holmgren J. The adjuvant effect of *Vibrio cholerae* and *Escherichia coli* heat-labile enterotoxins is linked to their ADP-ribosyltransferase activity. European journal of immunology. 1992;22(9):2277–81. Epub 1992/09/01. 10.1002/eji.1830220915 . 1381311

[41] Agren LC, Ekman L, Lowenadler B, Lycke NY. Genetically engineered nontoxic vaccine adjuvant that combines B cell targeting with immunomodulation by cholera toxin A1 subunit. J Immunol. 1997;158(8):3936–46. . 9103464

[42] Eriksson A, Lycke N. The CTA1-DD vaccine adjuvant binds to human B cells and potentiates their T cell stimulating ability. Vaccine. 2003;22(2):185–93. Epub 2003/11/15. 10.1016/s0264-410x(03)00567-x . 14615145

[43] Bowman CC, Clements JD. Differential biological and adjuvant activities of cholera toxin and Escherichia coli heat-labile enterotoxin hybrids. Infect Immun. 2001;69(3):1528–35. Epub 2001/02/17. 10.1128/IAI.69.3.1528-1535.2001 11179323 PMC98052

[44] Farquhar MJ, Harris HJ, Diskar M, Jones S, Mee CJ, Nielsen SU, et al Protein kinase A-dependent step(s) in hepatitis C virus entry and infectivity. J Virol. 2008;82(17):8797–811. Epub 2008/06/27. 10.1128/JVI.00592-08 18579596 PMC2519651

[45] Liu D, Guo H, Zheng W, Zhang N, Wang T, Wang P, et al Discovery of the cell-penetrating function of A2 domain derived from LTA subunit of Escherichia coli heat-labile enterotoxin. Appl Microbiol Biotechnol. 2016;100(11):5079–88. 10.1007/s00253-016-7423-x . 26960316

[46] Norton EB, Branco LM, Clements JD. Evaluating the A-Subunit of the Heat-Labile Toxin (LT) As an Immunogen and a Protective Antigen Against Enterotoxigenic Escherichia coli (ETEC). PLoS One. 2015;10(8):e0136302 10.1371/journal.pone.0136302 26305793 PMC4549283

[47] Roederer M, Nozzi JL, Nason MC. SPICE: exploration and analysis of post-cytometric complex multivariate datasets. Cytometry A. 2011;79(2):167–74. Epub 2011/01/26. 10.1002/cyto.a.21015 21265010 PMC3072288

