# Supplementary figures and images for: LTA1 and dmLT enterotoxin-based proteins activate antigen-presenting cells independent of PKA and despite distinct cell entry mechanisms
Source: PLoS One. 2020 Jan 13;15(1):e0227047. doi: 10.1371/journal.pone.0227047 (PMC6957164; doi:10.1371/journal.pone.0227047)

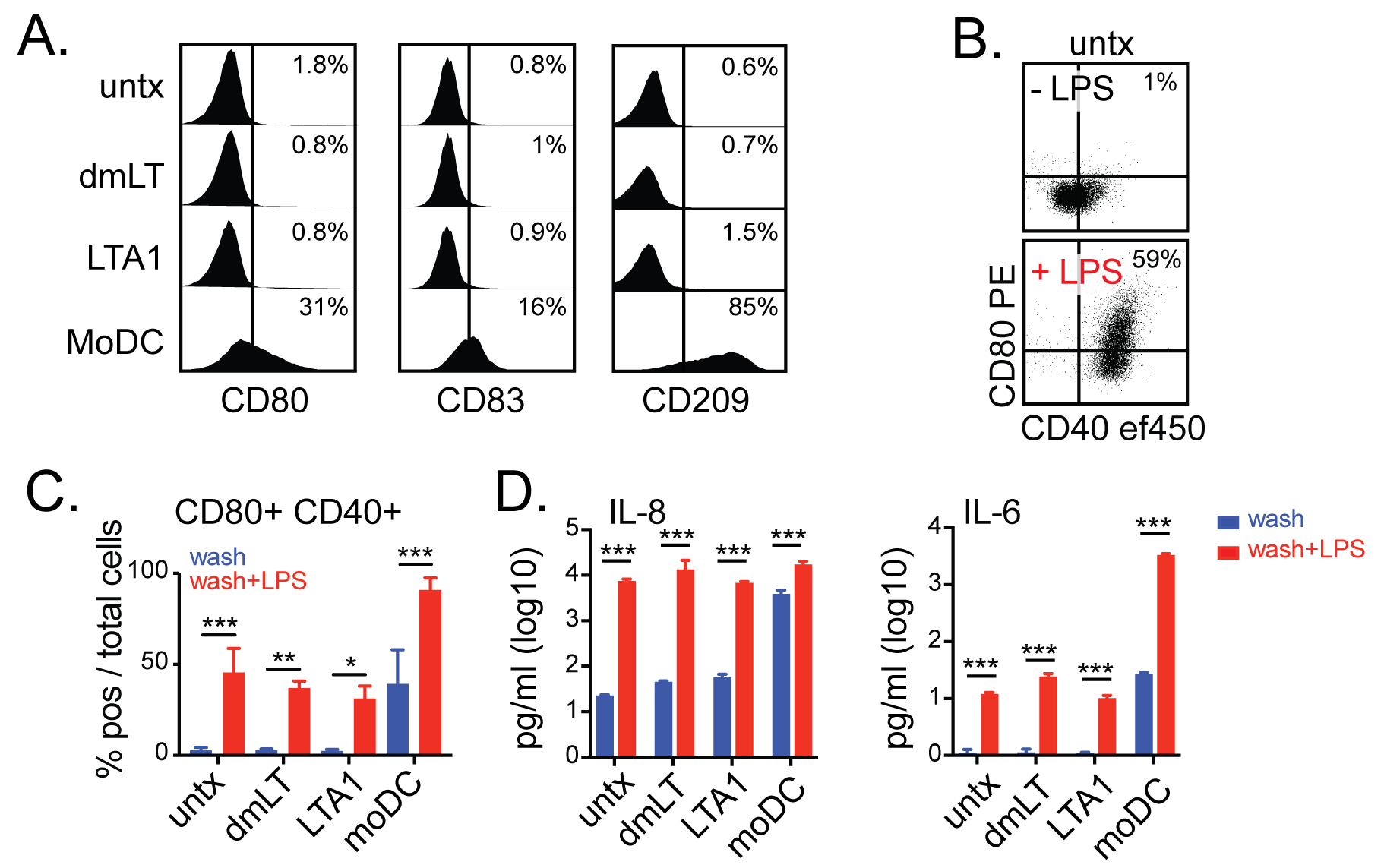

Supplement: S1 Fig — To evaluate changes to APCs, THP-1 cells were treated with media alone (untx), with 1 μg/ml dmLT or 10μg/ml LTA1, or ionomycin and cytokine (IL-4, GM-CSF, TNF-α) to induce differentiation into dendritic cells (moDC). After treatment, cells were incubated in fresh media with or without 10ng/ml LPS for 24h. Cytometric samples and experiments were performed in triplicate; cytokine analyses were performed with triplicate samples. (A) Representative histograms for costimulatory molecules CD80, CD83, and CD209. (B) Representative CD40 vs CD80 dot plots from untx cells with or without LPS stimulation. (C) Mean+SEM positive cells gated for total cells. Significance tested by two-way ANOVA with Bonferroni post-test between selected pairs as indicated (*P ≤ 0.05, **P ≤ 0.01, ***P ≤ 0.001). Bars at mean+SEM. (TIF) [file pone.0227047.s001.tif]

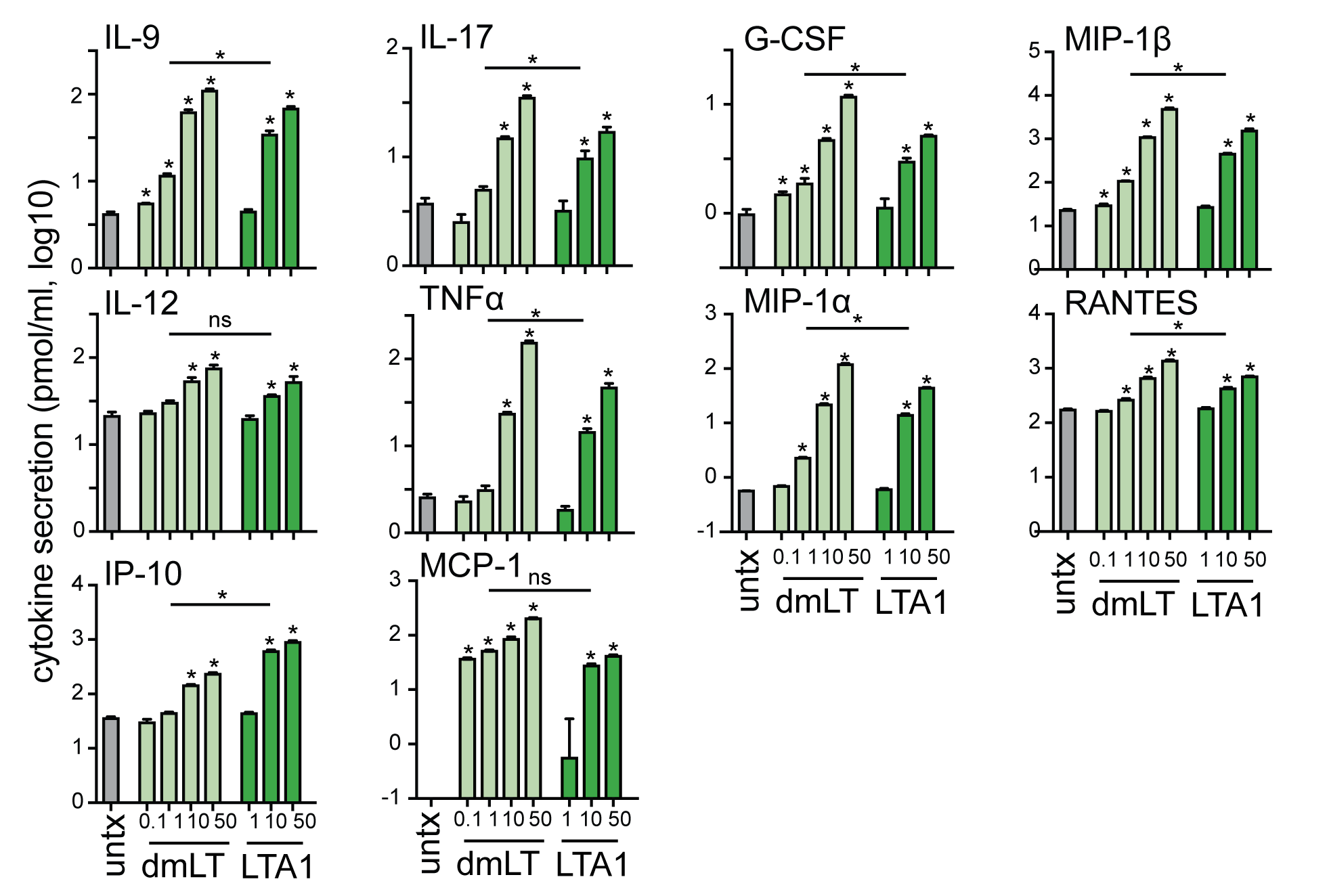

Supplement: S2 Fig — To evaluate changes to APCs, THP-1 cells were treated with media alone (untx) or with dmLT or LTA1 in μg doses/ml indicated or 10 ng/ml PMA (Mϕ). Cytokine analyses were performed with triplicate samples. Selected mean+SEM secreted cytokines after 24h culture detected by Human 27-plex Bioplex are shown. Significance tested by ANOVA with Bonferroni post-test for all groups compared to untx and as indicated (*P ≤ 0.05). (TIF) [file pone.0227047.s002.tif]

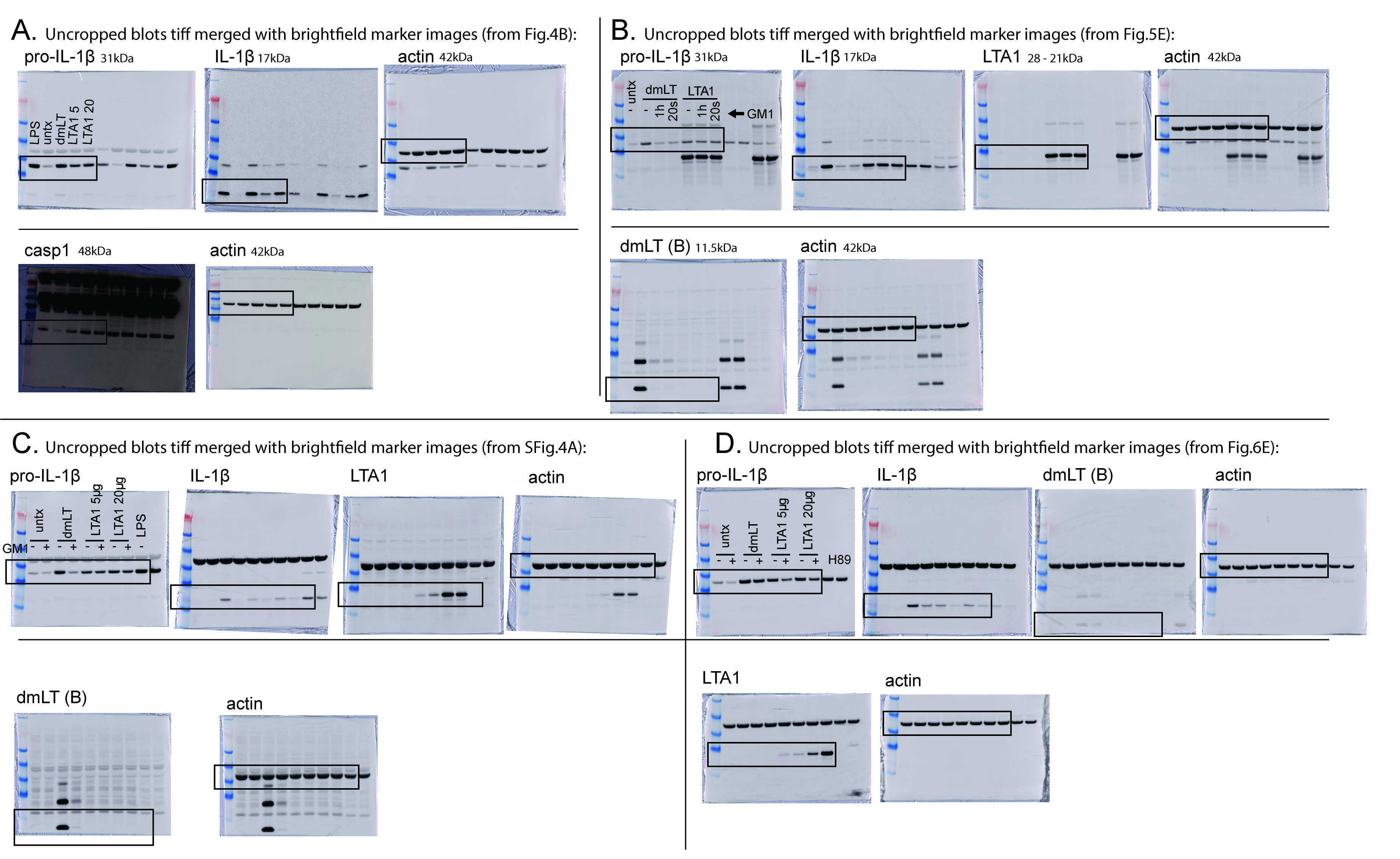

Supplement: S3 Fig — Uncropped jpg ECL images of Western blots merged with brightfield images showing colorimetric standard SeeBlue Plus 2 and detection antibody indicated on top of image. Rectangle selections indicate cropped images used in Fig 4B (A), Fig 5E (B), S4A Fig Supplemental (C) and Fig 6E (D). (TIF) [file pone.0227047.s003.tif]

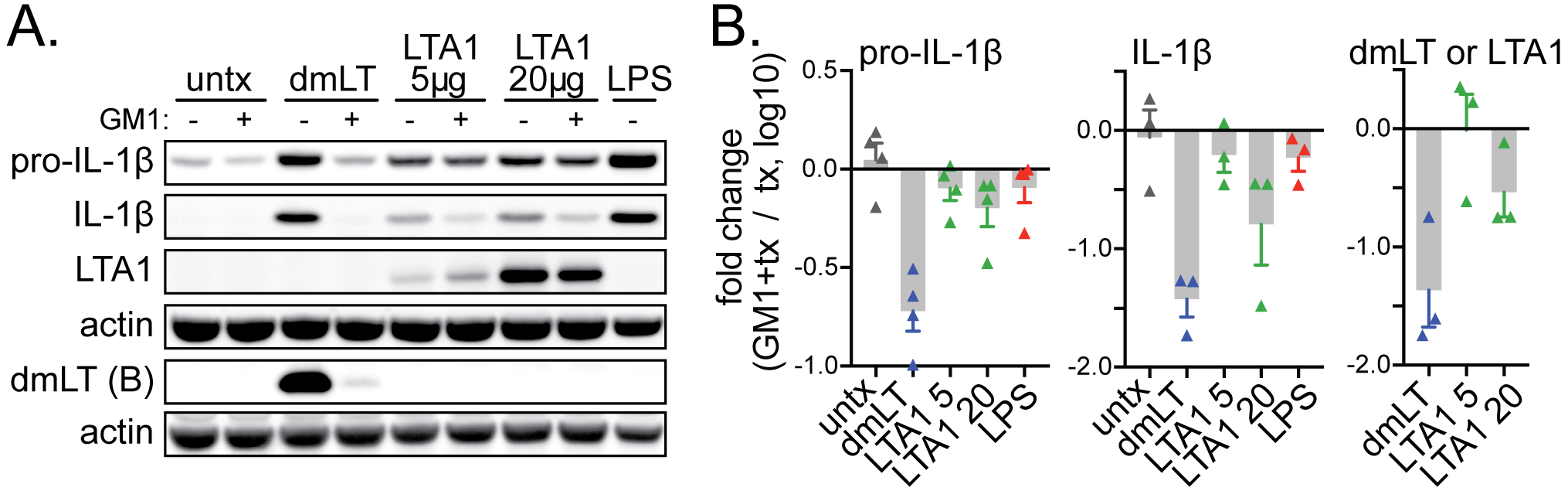

Supplement: S4 Fig — THP-1 cells (0.5e6/ml) were incubated with PMA for 12 h then left untreated (untx) or stimulated for 12h with positive control 1 μg/ml LPS, 0.5 μg/ml dmLT, or 5–20 μg/ml LTA1 as indicated. Experiments performed at least in triplicate. In some cases, treatments were pre-incubated with GM1 for 15 min at 20C prior to cell treatments. (A) Representative Western blots images for indicated protein bands using lysates of THP-1 cells. (B) Fold change of GM1+treatment from treatment using relative intensity of protein bands normalized to actin compiled from 3 or more separate experiments. Bars at mean+SEM. (TIF) [file pone.0227047.s004.tif]
